# Supplementary material for: DNA methylation signatures of monozygotic twins clinically discordant for multiple sclerosis
Source: Nat Commun. 2019 May 7;10:2094. doi: 10.1038/s41467-019-09984-3 (PMC6504952; doi:10.1038/s41467-019-09984-3)
Supplement: Supplementary file 1 — Supplementary Information [file 41467_2019_9984_MOESM1_ESM.pdf]

Supplementary Information:

DNA methylation signatures of monozygotic twins clinically discordant for multiple sclerosis

Souren et al.

Supplementary Tables

Supplementary Table 1. Characteristics of the top 15 most significantly differentially methylated positions (DMPs) associated with long-standing MS, identified by a pair-wise analysis including only the EPIC array data of the 25 MZ twins pairs that have been clinically discordant for MS for more than 10 years (n = 25 twin pairs). The first two DMPs had a suggestive P-value<$5*10^{-6}$ (adjusted for cell type composition).

						Mean β- value (U)					
	Probe ID	Location[a]	Gene	Functional region[b]	450k	MS-affected co-twins (n=25)	non-affected co-twins (n=25)	Mean Δβ-value (95% CI) (U)	Mean Δβ-value (95% CI) (A)	β-value range	$P_{W\text{-}u}/P_{W\text{-}A}$
1	**cg11243634**	chr1:59044320	***TACSTD2***	TSS1500/DHS	Y	0.667	0.689	-0.022 (-0.029,-0.015)	-0.022 (-0.029,-0.015)	0.62-0.73	$\mathbf{1.13*10^{-6}/2.56*10^{-6}}$
2	**cg23896094**	chr9:4839083	***RCL1***	TSS1500/Body/ TFBS	N	0.890	0.901	-0.011 (-0.016,-0.007)	-0.011 (-0.016,-0.007)	0.87-0.93	$\mathbf{4.17*10^{-6}/4.17*10^{-6}}$
3	cg06958567	chr18:52495541	*RAB27B*	TSS1500/DHS	Y	0.120	0.129	-0.009 (-0.015,-0.004)	-0.010 (-0.015,-0.006)	0.08-0.18	$3.29*10^{-4}/8.17*10^{-6}$
4	cg07850221	chr19:36235109	*U2AF1L4/ PSENEN*	Body/TSS1500/ TFBS	Y	0.825	0.834	-0.009 (-0.012,-0.005)	-0.009 (-0.013,-0.005)	0.78-0.87	$1.83*10^{-5}/8.17*10^{-6}$
5	cg22891413	chr5:1407569	*SLC6A3*	Body	Y	0.901	0.910	-0.009 (-0.013,-0.005)	-0.009 (-0.013,-0.006)	0.85-0.94	$2.50*10^{-4}/1.01*10^{-5}$
6	cg04669407	chr5:148521450	*ABLIM3*	5'UTR/CpG island/DHS	Y	0.377	0.404	-0.027 (-0.037,-0.017)	-0.025 (-0.036,-0.015)	0.28-0.48	$1.97*10^{-6}/1.23*10^{-5}$
7	cg17514766	chr19:39826927	*GMFG*	TSS1500/TFBS	Y	0.105	0.115	-0.010 (-0.014,-0.005)	-0.010 (-0.014,-0.006)	0.08-0.16	$3.81*10^{-5}/1.83*10^{-5}$
8	cg22008026	chr4:155413392	*DCHS2*	TSS1500/CpG island/DHS	Y	0.213	0.237	-0.024 (-0.031,-0.016)	-0.021 (-0.029,-0.014)	0.16-0.31	$1.01*10^{-5}/1.83*10^{-5}$
9	**cg26583412**	chr5:110062780	***TMEM232***	TSS1500/DHS	N	0.528	0.490	0.038 (0.022,0.054)	0.038 (0.023, 0.052)	0.26-0.68	$5.39*10^{-5}/1.83*10^{-5}$
10	cg26630171	chr19:3459270	*NFIC*	Body/DHS	Y	0.906	0.897	0.008 (0.004,0.012)	0.008 (0.005,0.012)	0.86-0.93	$1.20*10^{-4}/1.83*10^{-5}$
11	cg23516613	chr19:12595698	*ZNF709*	TSS200/CpG island/DHS	Y	0.121	0.131	-0.010 (-0.014,-0.006)	-0.009 (-0.013,-0.006)	0.10-0.16	$1.01*10^{-5}/2.21*10^{-5}$
12	cg16468417	chr3:35835606	*ARPP-21*	3'UTR	Y	0.788	0.769	0.019 (0.011-0.026)	0.016 (0.009,0.022)	0.73-0.84	$3.19*10^{-5}/2.66*10^{-5}$
13	**cg27037608**	chr5:110062618	***TMEM232***	TSS200/TFBS	N	0.498	0.472	0.026 (0.014,0.038)	0.027 (0.015,0.038)	0.34-0.59	$8.80*10^{-5}/3.19*10^{-5}$
14	cg11079989	chr1:17222715	*CROCC*	Body/DHS	Y	0.135	0.148	-0.013 (-0.019,-0.008)	-0.013 (-0.018,-0.008)	0.11-0.19	$2.66*10^{-5}/4.54*10^{-5}$
15	cg10344516	chr10:6689948		DHS	N	0.152	0.14	0.012 (0.007,0.018)	0.012 (0.006,0.018)	0.10-0.20	$1.23*10^{-5}/5.39*10^{-5}$
756	**cg25345365**	chr11:114050114	***ZBTB16***	Body/DHS/ enhancer	N	0.526	0.566	-0.040 (-0.065,-0.015)	-0.036 (-0.057,-0.014)	0.36-0.72	0.002/0.0018

Source data are provided as a Source Data file. [a]All the genome coordinates are based on human genome build GRCh37/hg19. [b]Based on information provided by the Illumina manifest. Since all genes have multiple transcripts, the "UCSC_RefGene_Group" gene-related location is listed. A = adjusted for cell-type composition, CI = confidence interval, DHS = DNAse I hypersensitive site, n = number of MS-discordant MZ twin pairs, $P_{W\text{-}A}$ = P-value two-tailed Wilcoxon signed-rank test adjusted for cell-type composition, $P_{W\text{-}U}$ = P-value two-tailed Wilcoxon signed-rank test unadjusted for cell-type composition, TFBS = transcription factor binding site, TSS200 = the region from transcription start site (TSS) to −200 nt upstream of TSS, TSS1500 = −200 to −1500 nt upstream of TSS, U = unadjusted for cell-type composition, 450k = probe present on the 450k array (Y = yes, N = no), 5'UTR= 5' untranslated region, Δβ-value = within-pair β-value difference (clinically MS-affected MZ co-twin – non-affected MZ co-twin).

Supplementary Table 2. Evaluation of the *TMEM232* MS-DMPs in blood-based 450K EWAS data of 140 MS patients and 139 unrelated controls from Kular et al[1]. The results of the PBMC-based EPIC array EWAS in the 45 MZ twins clinically discordant for MS are shown as well.

			**Blood-based 450K EWAS of unrelated cases and controls**							**PBMC-based EPIC EWAS of MS discordant MZ twins**					
			Mean β-value (± SD)							Mean β -value					
Probe ID[a]	Location at chr 5:		MS patients (n=140)	Controls (n=139)	Mean Δβ-value	β-value range	P_u[b]	P_A[b]	P_{A2}[b]	MS-affected co-twins (n=45)	Non-affected co-twins (n=45)	Mean Δβ-value (95% CI)	β-value range	P_{W-U}	P_{W-A}
cg23279021	110062343	1st exon	0.309 ± 0.076	0.297 ± 0.084	0.012	0.11-0.67	0.20	0.13	0.24	0.320	0.295	0.025 (0.011,0.040)	0.08-0.49	0.001	$2.6*10^{-4}$
cg17248924	110062384	TSS200	0.327 ± 0.098	0.314 ± 0.107	0.012	0.12-0.76	0.25	0.15	0.23	0.457	0.444	0.013 (-0.005,0.031)	0.22-0.62	0.16	0.08
cg11641395	110062398	TSS200	0.276 ± 0.080	0.268 ± 0.086	0.008	0.10-0.55	0.32	0.22	0.35	0.437	0.425	0.012 (-0.001,0.025)	0.26-0.58	0.16	0.12
cg06429214	110062417	TSS200	0.276 ± 0.099	0.259 ± 0.108	0.017	0.05-0.76	0.12	0.07	0.19	0.309	0.284	0.025 (0.008,0.041)	0.09-0.50	0.008	0.005
cg25259944	110062473	TSS200	0.325 ± 0.089	0.308 ± 0.099	0.017	0.11-0.70	0.10	0.05	0.16	0.483	0.455	0.028 (0.012,0.044)	0.27-0.61	0.001	0.002
cg22429640	110062570	TSS200								0.539	0.514	0.025 (0.009,0.041)	0.33-0.65	0.005	0.003
cg19398821	110062608	TSS200								0.507	0.484	0.023 (0.006,0.041)	0.29-0.68	0.017	0.016
cg27037608	110062618	TSS200								0.488	0.466	0.022 (0.012,0.032)	0.31-0.59	$4.8*10^{-5}$	$4.3*10^{-6}$
cg17946588	110062682	TSS1500								0.461	0.439	0.022 (0.007,0.037)	0.26-0.60	0.006	0.005
cg10597099	110062725	TSS1500								0.544	0.522	0.022 (0.002,0.041)	0.22-0.73	0.032	0.013
cg19526166	110062729	TSS1500	0.388 ± 0.098	0.374 ± 0.112	0.014	0.15-0.80	0.23	0.14	0.18	0.532	0.505	0.027 (0.007,0.048)	0.31-0.71	0.013	0.007
cg26583412	110062780	TSS1500								0.516	0.482	0.034 (0.020,0.048)	0.23-0.68	$2.0*10^{-5}$	$1.3*10^{-5}$
cg06414816	110062837	TSS1500	0.403 ± 0.078	0.391 ± 0.087	0.012	0.20-0.69	0.21	0.15	0.31	0.547	0.526	0.021 (0.007,0.035)	0.35-0.64	0.010	0.010

Source data are provided as a Source Data file. [a]The 450K array contains only 7 of the 13 *TMEM232* promoter probes that are present on the EPIC array. [b]Significance estimated using linear regression (see Methods for details). CI = confidence interval, n = number of individuals, P_U = P-value unadjusted, P_A = P-value adjusted for sex, age and smoking status, P_{A2} = P-value adjusted for sex, age, smoking status and cell type composition, P_{W-A} = P-value Wilcoxon two-tailed signed-rank test adjusted for cell-type composition, P_{W-U} = P-value Wilcoxon two-tailed signed-rank test unadjusted for cell-type composition**,** TSS200 = the region from transcription start site (TSS) to −200 nt upstream of TSS, TSS1500 = −200 to −1500 nt upstream of TSS, Δβ-value = within-pair β-value difference (clinically MS-affected MZ co-twin – non-affected MZ co-twin).

Supplementary Table 3. MS-associated differentially methylated regions (MS-DMRs) identified in whole genome bisulfite sequencing (WBGS) data of CD4+ memory T cells of four MS discordant female MZ twin pairs (n = 4 twin pairs). MS-DMRs listed in this table were defined as ≥3 CpGs, each having P<0.05 (two-tailed paired T-test) and absolute mean methylation difference >0.15, and a maximum of 500 bp distance between neighbouring significant CpGs.

						Mean methylation						
Chr[a]	Start	End	Width	# CG	Location	MS-affected MZ co-twins	Non-affected MZ co-twins	Mean methylation difference	Gene	Full gene name	Robust DMR	Distance to closest EWAS EPIC probe and P_{W-A}[b]
1	17711436	17712116	681	3	Intron	0.74	0.91	-0.17	*PADI6*	Peptidyl arginine deiminase 6	No	900 bp, P>0.05
1	85791031	85791047	17	5	Intron	0.85	0.67	0.18	*DDAH1*	Dimethylarginine dimethylaminohydrolase 1[2,3]	Yes	4 kb
1	87644875	87644911	37	5	Distal Intergenic	0.56	0.76	-0.20			Yes	1 kb
1	228755378	228755464	87	3	Distal Intergenic	0.71	0.51	0.20			Yes	1.5 kb
2	87568953	87569230	278	3	Intron	0.40	0.59	-0.18	*RMND5A*	Required for meiotic nuclear division 5 homolog A	No	**0 bp** (cg21997198), P>0.05
5	176007126	176007142	17	3	Intron	0.75	0.57	0.18	*CDHR2*	Cadherin related family member 2	Yes	600 bp, P>0.05
6	57421688	57421697	10	3	Intron	0.71	0.51	0.20	*PRIM2*	DNA primase subunit 2	Yes	9 kb
6	170403829	170404107	279	3	Distal Intergenic	0.62	0.84	-0.22			Yes	250 bp, P>0.05
7	31117742	31118094	353	3	Intron	0.65	0.82	-0.17	*ADCYAP1R1*	ADCYAP receptor type I	No	1.5 kb
7	98424232	98424350	119	3	Distal Intergenic	0.51	0.70	-0.20			No	**0 bp** (cg16446288[c]/ cg11757417), P=0.04/ 0.86, Δβ=-0.01/0.00
8	102904079	102904097	19	3	Intron	0.33	0.53	-0.19	*NCALD*	Neurocalcin delta	Yes	170 bp, P>0.05
9	43134844	43135115	272	3	Promoter (1-2kb)	0.45	0.62	-0.17	*ANKRD20A3*	Ankyrin repeat domain 20 family member A3	Yes	90 bp, P>0.05
9	66493004	66493351	348	3	Promoter (1-2kb)	0.42	0.24	0.18	*PTGER4P2-CDK2AP2P2*	PTGER4P2-CDK2AP2P2 read through, transcribed pseudogene	No	**0 bp** (cg17548900), P>0.05
11	133519484	133519982	499	3	Distal Intergenic	0.35	0.60	-0.25			No	10 kb
13	27295982	27296125	144	3	Distal Intergenic	0.20	0.43	-0.23			Yes	**0 bp** (cg16557370[d]/ cg08419873), P>0.05
14	101291034	101291083	50	3	Promoter (1-2kb)	0.45	0.64	-0.19	*MEG3*	Maternally expressed 3 (non-protein coding)	Yes	**0 bp** (cg23870378), P>0.05
15	21083039	21083557	519	3	Distal Intergenic	0.62	0.42	0.20			No	50 kb
16	22545670	22545683	14	3	Exon	0.59	0.80	-0.21	*NPIPB5*	Nuclear pore complex interacting protein family member B5	Yes	5 kb
20	23515851	23516134	284	3	Intron	0.67	0.84	-0.17	*CST13P*	Cystatin 13, pseudogene	No	4 kb
**X[e]**	**130863481**	**130863509**	**29**	**3**	**Intron**	**0.66**	**0.40**	**0.26**	***FIRRE***	**Firre intergenic repeating RNA element**	Yes	**6 bp (cg08117231), P>0.05**

Source data are provided as a Source Data file. [a]Genomic coordinates are based on human genome build GRCh37/hg19. The MS-DMRs were annotated using the ChIPseeker R/Bioconductor package (v1.14.2)[4]. [b]In this column the approximate distance to the closest EWAS EPIC probe is listed. When the distance is <1 kb, then of this EPIC probe the P_{W-A}-value of the pair-wise analysis using the EPIC array data of the 45 MZ twin pairs adjusted for cell-type composition is listed as well. If the distance is 0 bp, then the EPIC probe is located within the MS-DMR. [c]cg16446288 is exactly located at chr7:98424232-98424233. [d]cg16557370 is

exactly located at chr13:27295982-27295983. [e]This MS-DMR fulfilled the stringent selection criteria of ≥3 CpGs, each having $P<0.05$ (two-tailed paired T-test) and absolute mean methylation difference >0.20, and a maximum of 500 bp distance between neighbouring significant CpGs. P_{W-A} = P-value two-tailed Wilcoxon signed-rank test adjusted for cell-type composition. Robust DMR = DMR that shows overall consistent methylation differences (same direction) across the entire DMR (applying the lower methylation threshold of 0.15 resulted in several MS-DMRs not showing consistent methylation differences (same direction) across the entire DMR). Please see the Source Data File for details. $\Delta\beta$ = Within-pair β-value difference (clinically MS-affected MZ co-twin – non-affected MZ co-twin).

Supplementary Table 4. Within-pair differentially methylated regions (WP-DMRs)[a] identified in the EPIC array data of the 45 MZ twins clinically discordant for multiple sclerosis (MS).

Gene locus	Chr	IR	Location first CpG[b]	Location last CpG[b]	#EPIC probes	#Twin pairs	Abnormal methylation profile	Methylation aberration	Pair	Treat-ment
RBP7	1		10057303	10057312	3	1	Non-affected co-twin	Hyper	E	GLAT
KIF26B	1		245710332	245710401	3	1	MS co-twin	Hypo	AG	IFN
PAX8-AS1/PAX8/ LOC440839/LOC654433	2		113992694	113993313	7	1	MS co-twin	Hypo	V	DMF
DUSP19	2		183943175	183943698	9	1	MS co-twin	Hyper	AD	IFN
PLOD2	3		145878963	145878979	3	1	MS co-twin	Hyper	AN	IFN
LRRC34	3		169531663	169531783	3	1	MS co-twin	Hyper	AD	IFN
RP11-1398P2.1	4		1581921	1582181	4	1	MS co-twin	Hyper	V	DMF
TACR3	4		104640662	104641250	4	1	MS co-twin	Hyper	G	
PCDH10	4		134070433	134070441	3	1	MS co-twin	Hyper	Y	IFN
PCDHG gene cluster	5		140749783	140750160	4	1	MS co-twin	Hyper	P	
PCDHG gene cluster	5		140762261	140762315	3	1	MS co-twin	Hyper	P	
PCDHG gene cluster	5		140792511	140792540	3	1	MS co-twin	Hyper	P	
PCDHG gene cluster	5		140810051	140810137	3	1	MS co-twin	Hyper	P	
DPYSL3	5		146889238	146889275	3	1	Non-affected co-twin	Hyper	AA	IFN
CCNG1	5		162864291	162864633	8	1	MS co-twin	Hyper	Y	IFN
HIST1H3E	6		26224013	26224925	6	2	Non-affected co-twins	Hyper	H/AG	TFM/IFN
HIST1H2AL	6		27833095	27833555	3	1	Non-affected co-twin	Hyper	U	IFN
NA	6		30434109	30434324	5	1	MS co-twin	Hyper	V	DMF
AGPAT1/RNF5/RNF5P1	6		32146466	32146595	4	1	MS co-twin	Hyper	P	
DNAH8	6		38682995	38683221	4	1	MS co-twin	Hyper	AD	IFN
SVOPL	7	Y	138348774	138349443	5	1	Non-affected co-twin	Hypo	AB	IFN
NA	7		158750244	158751184	5	1	MS co-twin	Hyper	V	DMF
DLC1	8		13134144	13134166	3	1	Non-affected co-twin	Hyper	R	
TRMT12	8		125462982	125463066	4	1	MS co-twin	Hyper	W	
NEBL-AS1/NEBL	10		21462747	21462768	3	1	Non-affected co-twin	Hyper	AA	IFN
HSD17B7P2	10		38645376	38645740	3	1	Non-affected co-twin	Hyper	AD	IFN
CAT	11		34460140	34460557	3	1	MS co-twin	Hyper	H	TFM
DIXDC1	11		111847892	111848326	3	1	Non-affected co-twin	Hyper	AA	IFN
WDR66	12		122356316	122356598	5	1	Non-affected co-twin	Hyper	AD	IFN
GPR133	12		131488390	131488726	3	1	MS co-twin	Hypo	AD	IFN
DHRS4L2	14		24438909	24439192	4	1	Non-affected co-twin	Hyper	AD	IFN
CLEC14A	14		38724646	38724675	3	1	MS co-twin	Hyper	W	
PAK6/C15orf56	15		40545050	40545145	3	1	Non-affected co-twin	Hyper	E	GLAT
LOC101928414/CTD-2651B20.3	15		45571526	45571636	4	1	MS co-twin	Hyper	AD	IFN
NA	15		53092788	53093509	3	1	MS co-twin	Hyper	AG	IFN
CLK3	15		74890733	74891207	3	1	Non-affected co-twin	Hyper	AD	IFN
UNC45A	15		91473167	91473569	6	1	Non-affected co-twin	Hyper	P	
ITGAM	16		31342453	31343056	4	1	Non-affected co-twin	Hyper	T	
C17orf97	17		259755	259924	3	1	MS co-twin	Hyper	AG	IFN
L3MBTL4	18		6414958	6414978	4	1	Non-affected co-twin	Hyper	AA	IFN
ZNF254	19		24269919	24270468	4	1	MS co-twin	Hyper	AD	IFN
LYPD5	19		44324903	44325004	3	1	MS co-twin	Hyper	AD	IFN
ISOC2	19		55972646	55973778	11	2	MS & Non-affected co-twin	Hyper	H/BA	TFM/-
AURKC	19		57742345	57742423	4	1	MS co-twin	Hypo	AG	IFN
HM13/MCTS2P	20	Y	30134929	30135362	7	1	Non-affected co-twin	Hyper	B	IFN

Source data are provided as a Source Data file. [a]WP-DMRs were defined as ≥3 CpGs with a within-pair β-value difference >0.20 (adjusted for cell-type composition) and a maximum 1 kb distance between neighboring CpGs (the 257 IFN-associated CpGs were excluded from this analysis). In addition, the β-value of the "abnormally methylated" co-twin had to be greater than ±3 standard deviations from the mean. [b]All genome coordinates are based on human genome build GRCh37/hg19. Chr = chromosome, DMF = dimethyl fumarate, GLAT = glatiramer acetate, IFN = interferon-beta, IR = imprinted region (Y = yes), TFM = teriflunomide.

Supplementary Table 5. Results of the evaluation whether the 27 WP-DMRs[a], that were aberrantly methylated in the MS-affected co-twins (listed in Table 4), were present in other pairs as well by applying a lower Δβ-value threshold of 0.15. In total, four WP-DMRs were also identified in other twin pairs, of which one intergenic WP-DMR was present in 4 pairs and always associated with the MS phenotype (in bold). Hence, in total 24 MS-associated WP-DMRs were identified in 11 pairs, of which 23 were pair-specific and one present in 4 twin pairs. Clinical characteristics such as gender, MS course, disease duration at sampling date, age at first disease manifestation, MS treatment, and pack-years at sample collection did not differ between these 11 twin pairs and the 34 other pairs (P>0.05, two-tailed Wilcoxon rank sum test for continuous data and two-tailed Fisher's exact test for categorical data).

Gene locus	Chr	IR	Location first CpG[b]	Location last CpG[b]	#EPIC probes	#Twin pairs	Abnormal methylation profile	Methylation aberration	Pair	Treat-ment	**#Twin pairs Δβ-value 0.15[c]**	Abnormal methylation profile	Pair[d]	Treatment
KIF26B	1		245710332	245710401	3	1	MS co-twin	Hypo	AG	IFN	1	MS co-twin	AG	IFN
PAX8-AS1/PAX8/ LOC440839/LOC654433	2		113992694	113993313	7	1	MS co-twin	Hypo	V	DMF	2	Non-affected co-twin & MS co-twin	U/V	IFN/DMF
DUSP19	2		183943175	183943698	9	1	MS co-twin	Hyper	AD	IFN	1	MS co-twin	AD	IFN
PLOD2	3		145878963	145878979	3	1	MS co-twin	Hyper	AN	IFN	1	MS co-twin	AN	IFN
LRRC34	3		169531663	169531783	3	1	MS co-twin	Hyper	AD	IFN	1	MS co-twin	AD	IFN
RP11-1398P2.1	4		1581921	1582181	4	1	MS co-twin	Hyper	V	DMF	3	MS co-twin & Non-affected co-twin	L/V/AF	-/DMF/NAT
TACR3	4		104640662	104641250	4	1	MS co-twin	Hyper	G		1	MS co-twin	G	
PCDH10	4		134070433	134070441	3	1	MS co-twin	Hyper	Y	IFN	1	MS co-twin	Y	IFN
PCDHG gene cluster	5		140749783	140750160	4	1	MS co-twin	Hyper	P		1	MS co-twin	P	
PCDHG gene cluster	5		140762261	140762315	3	1	MS co-twin	Hyper	P		1	MS co-twin	P	
PCDHG gene cluster	5		140792511	140792540	3	1	MS co-twin	Hyper	P		1	MS co-twin	P	
PCDHG gene cluster	5		140810051	140810137	3	1	MS co-twin	Hyper	P		1	MS co-twin	P	
–CCNG1	5		162864291	162864633	8	1	MS co-twin	Hyper	Y	IFN	1	MS co-twin	Y	IFN
NA	6		30434109	30434324	5	1	MS co-twin	Hyper	V	DMF	1	MS co-twin	V	DMF
AGPAT1/RNF5/RNF5P1	6		32146466	32146595	4	1	MS co-twin	Hyper	P		1	MS co-twin	P	
DNAH8	6		38682995	38683221	4	1	MS co-twin	Hyper	AD	IFN	1	MS co-twin	AD	IFN
**NA**	**7**		**158750244**	**158751184**	**5**	**1**	**MS co-twin**	**Hyper**	**V**	**DMF**	**4**	**MS co-twin**	**U/V/AB /AH**	**IFN/DMF/ IFN/IFN**
TRMT12	8		125462982	125463066	4	1	MS co-twin	Hyper	W		1	MS co-twin	W	
CAT	11		34460140	34460557	3	1	MS co-twin	Hyper	H	TFM	2	MS co-twin & Non-affected co-twin	H/P	TFM/-
GPR133	12		131488390	131488726	3	1	MS co-twin	Hypo	AD	IFN	1	MS co-twin	AD	IFN
CLEC14A	14		38724646	38724675	3	1	MS co-twin	Hyper	W		1	MS co-twin	W	
LOC101928414/CTD-2651B20.3	15		45571526	45571636	4	1	MS co-twin	Hyper	AD	IFN	1	MS co-twin	AD	IFN
NA	15		53092788	53093509	3	1	MS co-twin	Hyper	AG	IFN	1	MS co-twin	AG	IFN
C17orf97	17		259755	259924	3	1	MS co-twin	Hyper	AG	IFN	1	MS co-twin	AG	IFN
ZNF254	19		24269919	24270468	4	1	MS co-twin	Hyper	AD	IFN	1	MS co-twin	AD	IFN
LYPD5	19		44324903	44325004	3	1	MS co-twin	Hyper	AD	IFN	1	MS co-twin	AD	IFN
AURKC	19		57742345	57742423	4	1	MS co-twin	Hypo	AG	IFN	1	MS co-twin	AG	IFN

Source data are provided as a Source Data file. [a]WP-DMRs were defined as ≥3 CpGs with a within-pair β-value difference >0.20 (adjusted for cell-type composition) and a maximum 1 kb distance between neighboring CpGs (the 257 IFN-associated CpGs were excluded from this analysis). In addition, the β-value of the "abnormally methylated" co-twin had to be greater than ±3 standard deviations from the mean. [b]All genome coordinates are based on human genome build GRCh37/hg19. [c]A Δβ-value threshold of 0.15 was used to evaluate whether the 27 WP-DMRs, that were aberrantly methylated in the MS-affected co-twins, were present in other twin pairs as well. Chr = chromosome, DMF = dimethyl fumarate, GLAT = glatiramer acetate, IFN = interferon-beta, IR = imprinted region (Y = yes), TFM = teriflunomide.

Supplementary Table 6. The 25 differentially variable positions (DVPs) identified between MS-affected and clinically non-affected MZ co-twins using iEVORA (n = 45 twin pairs).[5]

	Probe ID	Location[a]	Gene	Functional region[b]	$P_{unpaired\ T\text{-}Test}$	$P_{FDR\text{-}corrected\ Barlett's\ test}$	Hypervariable Group
1	cg09319843	chr18:25757569	*CDH2*	TSS200/CpG Island	$1.49*10^{-03}$	$2.63*10^{-04}$	Non-affected
2	cg07380496	chr5:71403420	*MAP1B*	1stExon/CpG Island	$2.05*10^{-03}$	$3.08*10^{-07}$	Non-affected
3	cg08927443	chr18:25757565	*CDH2*	TSS200/CpG Island	$2.25*10^{-03}$	$1.62*10^{-07}$	Non-affected
4	cg21303011	chr3:24537177	*THRB*	TSS1500/CpG Island	$4.28*10^{-03}$	$1.81*10^{-04}$	Non-affected
5	cg11732619	chr5:168728076	*SLIT3*	5'UTR/1stExon/CpG Island	$4.92*10^{-03}$	$2.02*10^{-04}$	Non-affected
6	cg11181094	chr9:125093748			$6.07*10^{-03}$	$4.82*10^{-05}$	Non-affected
7	cg13913015	chr2:47797963	*KCNK12*	TSS1500/CpG Island	$9.64*10^{-03}$	$5.09*10^{-06}$	Non-affected
8	cg06090660	chr18:25757555	*CDH2*	TSS200/CpG Island	$1.17*10^{-02}$	$5.39*10^{-04}$	Non-affected
9	cg11777419	chr14:104604401	*KIF26A*	TSS1500/CpG Island	$1.20*10^{-02}$	$5.49*10^{-06}$	Non-affected
10	cg23526824	chr17:38245542	*THRA*	Body	$1.29*10^{-02}$	$5.22*10^{-04}$	Non-affected
11	cg26452004	chr14:69726546	*GALNT16*	TSS200/CpG Island	$1.57*10^{-02}$	$7.33*10^{-04}$	Non-affected
12	cg26330510	chr5:1155853			$1.62*10^{-02}$	$2.45*10^{-09}$	Non-affected
13	cg07147599	chr16:50502136			$1.88*10^{-02}$	$3.65*10^{-04}$	Non-affected
14	cg26245302	chr6:163148501	*PARK2/PACRG*	TSS1500/Body/5'UTR/CpG Island	$2.34*10^{-02}$	$8.30*10^{-06}$	Non-affected
15	cg09936645	chr1:207627581	*CR2*	TSS200/CpG Island	$2.43*10^{-02}$	$3.02*10^{-08}$	Non-affected
16	cg07848601	chr5:170289430	*RANBP17*	Body/CpG Island	$2.52*10^{-02}$	$4.81*10^{-04}$	Non-affected
17	cg08558397	chr7:752149	*PRKAR1B*	5'UTR/1stExon/CpG Island	$2.90*10^{-02}$	$9.26*10^{-06}$	Non-affected
18	cg23307163	chr10:4828732			$2.98*10^{-02}$	$4.68*10^{-04}$	Non-affected
19	cg16026114	chr1:232765417			$3.69*10^{-02}$	$2.16*10^{-06}$	Non-affected
20	cg25088874	chr4:95678817	*BMPR1B*	TSS1500/CpG Island	$4.19*10^{-02}$	$4.60*10^{-06}$	Non-affected
21	cg23683528	chr2:235860449	*SH3BP4*	TSS200/CpG Island	$4.35*10^{-02}$	$5.96*10^{-06}$	MS-affected
22	cg20928782	chr11:63803364	*MACROD1*	Body	$4.46*10^{-02}$	$2.22*10^{-04}$	MS-affected
23	cg12954230	chr15:100882231	*ADAMTS17*	TSS200/CpG Island	$4.55*10^{-02}$	$3.57*10^{-07}$	Non-affected
24	cg21947590	chr19:620162	*POLRMT*	Body/CpG Island	$4.58*10^{-02}$	$4.33*10^{-05}$	Non-affected
25	cg09272992	chr7:150497601	*TMEM176B*	5'UTR/TSS1500/1stExon/CpG Island	$4.58*10^{-02}$	$7.97*10^{-06}$	MS-affected

Source data are provided as a Source Data file. DVPs were defined as CpGs with a FDR-corrected Barlett's P-value<0.001 and raw T-test P-value<0.05. [a]All genome coordinates are based on human genome build GRCh37/hg19. [b]Based on information provided by the Illumina manifest.

Supplementary Table 7. Characteristics of the seven most significant interferon-beta-associated differentially methylated positions (IFN-DMPs) (absolute mean Δβ-value>0.10 and P_{W-U}<0.001), identified by a pair-wise analysis only including the EPIC array data of the 12 pairs of which the MS-affected co-twins were treated with IFN at the moment of blood collection (n = 12 twin pairs).

Probe ID	Location[a]	Gene	Functional region[b]	450k	Mean β-value (U/A) IFN-treated MS-affected co-twins	Mean β-value (U/A) non-affected co-twins	Mean Δβ-value (95% CI) (U)	Mean Δβ-value (95% CI) (A)	β-value range	P_{W-u}/P_{W-A}	Full name
cg03607951	chr1:79085586	*IFI44*[c]	TSS1500/DHS	Y	0.57/0.59	0.69/0.68	-0.12 (-0.16,-0.08)	-0.09 (-0.13,-0.05)	0.44-0.77	$9.77*10^{-4}$ /$1.46*10^{-3}$	Interferon-induced protein 44-like
cg06981309	chr3:146260954	*PLSCR1*	5'UTR/DHS	Y	0.59/0.60	0.71/0.70	-0.12 (-0.16,-0.07)	-0.09 (-0.13,-0.05)	0.50-0.76	$4.88*10^{-4}$ /$4.88*10^{-4}$	Phospholipid scramblase 1
cg10549986	chr2:7018153	*RSAD2*	1st exon/DHS	Y	0.17/0.18	0.31/0.30	-0.14 (-0.19,-0.09)	-0.12 (-0.16,-0.07)	0.11-0.44	$4.88*10^{-4}$ /$4.88*10^{-4}$	Radical S-adenosyl methionine domain-containing protein 2
cg10771443	chr2:7018855	*RSAD2*	Body/DHS	N	0.36/0.38	0.49/0.45	-0.13 (-0.18,-0.07)	-0.07 (-0.12,-0.03)	0.26-0.56	$9.77*10^{-4}$ /$9.27*10^{-3}$	
cg15839328	chr2:7018885	*RSAD2*	Body/DHS	N	0.36/0.38	0.49/0.47	-0.13 (-0.18,-0.08)	-0.09 (-0.12,-0.05)	0.27-0.57	$4.88*10^{-4}$ /$1.46*10^{-3}$	
cg21549285	chr21:42799141	*MX1*	5'UTR/DHS	Y	0.63/0.64	0.79/0.78	-0.16 (-0.22,-0.09)	-0.14 (-0.20,-0.07)	0.44-0.86	$4.88*10^{-4}$ /$1.46*10^{-3}$	MX dynamin like GTPase 1
cg26312951	chr21:42797847	*MX1*	TSS200/5'UTR/ TFBS/open chromatin	Y	0.32/0.33	0.43/0.42	-0.11 (-0.16,-0.07)	-0.09 (-0.13,-0.05)	0.17-0.48	$4.88*10^{-4}$ /$2.44*10^{-3}$	

Source data are provided as a Source Data file. [a]All the genome coordinates are based on human genome build GRCh37/hg19. [b]Based on information provided by the Illumina manifest. Since all genes have multiple transcripts, the "UCSC_RefGene_Group" gene-related location is listed. [c]Other potential IFN-DMPs in *IFI44L* are cg13452062 (Δβ-value = 0.20, P_{W-U} = 0.002) and cg05696877 (Δβ-value = 0.15, P_{W-U} = 0.001). 450k = probe present on the 450k array (Y = yes, N = no), A = adjusted for cell-type composition, CI = confidence interval, DHS = DNAse I hypersensitive site, IFN-DMPs = interferon-beta treatment-associated differentially methylated positions, n = number of pairs, P_{W-A} = P-value two-tailed Wilcoxon signed-rank test adjusted for cell-type composition, P_{W-U} = P-value two-tailed Wilcoxon signed-rank test unadjusted for cell-type composition, TFBS = transcription factor binding site, TSS200 = the region from transcription start site (TSS) to −200 nt upstream of TSS, TSS1500 = −200 to −1500 nt upstream of TSS, U = unadjusted for cell-type composition, 5'UTR= 5' untranslated region, Δβ = within-pair β-value difference (clinically MS-affected, IFN-treated MZ co-twin – non-affected MZ co-twin).

Supplementary Table 8. Estimated cell type proportions of the 12 pairs of which the clinically MS-affected MZ co-twins were treated with interferon-beta (IFN) at the moment of blood collection (n = 12 twin pairs).[a]

Cell Types	Cellular proportions IFN-treated MS-affected MZ co-twins[b]	Cellular proportions clinically non-affected MZ co-twins[b]	Mean within-pair difference(95% CI)[b]	P_W
CD4+ T cells	0.35 ± 0.08	0.34 ± 0.06	0.01 (-0.02,0.04)	0.57
CD8+ T cells	0.18 ± 0.08	0.19 ± 0.06	-0.01 (-0.04,0.03)	0.57
CD19+ B cells	0.14 ± 0.04	0.12 ± 0.03	0.02 (0.01,0.04)	**0.01**
CD14+ Monocytes	0.18 ± 0.07	0.19 ± 0.07	-0.01 (-0.04,0.02)	0.42
CD56+ NK cells	0.06 ± 0.07	0.12 ± 0.09	-0.06 (-0.10,-0.02)	**0.007**
Granulocytes	0.09 ± 0.12	0.04 ± 0.06	0.05 (-0.01,0.10)	0.09

Source data are provided as a Source Data file. [a]Cell type proportions were estimated using the DNA methylome reference-based method of Houseman et al.[6] implemented in the *minfi* R/Bioconductor package[7]. [b]Values are expressed as mean ± SD. [c]Within-pair difference = clinically MS-affected, IFN-treated MZ co-twin – non-affected MZ co-twin. CI = confidence interval, n = number of pairs. P_W=P-value nonparametric two-tailed Wilcoxon signed-rank test. P-values<0.05 are in **bold**.

Supplementary Table 9. Summary of the 41 GC-DMRs[a] (in 39 genes) that overlap with GC-response (dexamethasone) genes recorded in the EMBL-EBI Expression Atlas (accessed May 2018) (n =1 pair).

Gene	Chr	Start[b]	End[b]	Width	#CG	Location	Mean methylation GC-treated MS co-twin	Mean methylation unaffected co-twin	Mean methylation difference	Methylation aberration GC-treatment	Expression effect of GCs recorded in EMBL-EBI Expression Atlas
ADAMTS2	5	178652819	178652857	39	2	Intron	0.61	0.87	-0.26	Hypo	Up
ADAMTS2	5	178661985	178662010	26	3	Intron	0.85	0.60	0.25	Hyper	Up
ADORA3	1	112049050	112049096	47	2	Intron	0.64	0.90	-0.26	Hypo	Up
ALK	2	29794637	29794701	65	3	Intron	0.62	0.89	-0.27	Hypo	Up
APOBEC3A_B	22	39363192	39363237	46	2	Intron	0.88	055	0.33	Hyper	Down
ARRDC2	19	18124639	18124670	32	2	3' UTR	0.28	0.58	-0.29	Hypo	Up
ATP6V0D2	8	87126109	87126154	46	2	Exon	0.99	0.66	0.33	Hyper	Down
BCL11A	2	60756422	60756472	51	2	Intron	0.73	0.44	0.29	Hyper	Down
CALHM6	6	116783956	116783983	28	2	Intron	0.80	0.51	0.29	Hyper	Up
CCL26	7	75416004	75416035	32	2	Intron	0.61	0.88	-0.26	Hypo	Up
CD83	6	14122060	14122276	217	4	Intron	0.57	0.30	0.27	Hyper	Down
CDH1	16	68816259	68816295	37	2	Intron	0.51	0.05	0.45	Hyper	Down
COL4A2	13	110996989	110997025	37	3	Intron	0.67	0.92	-0.25	Hypo	Up
DDC	7	50597354	50597383	30	2	Intron	0.85	0.58	0.27	Hyper	Up
EGFR	7	55180338	55180378	41	2	Intron	0.52	0.84	-0.32	Hypo	Up
EVL	14	100524807	100524853	47	2	Intron	0.89	0.62	0.27	Hyper	Down
FAM49A	2	16745180	16745220	41	2	Intron	0.46	0.78	-0.32	Hypo	Up
FETUB	3	186367720	186367750	31	2	Intron	0.52	0.21	0.31	Hyper	Up
FGF18	5	170864899	170864934	36	2	Intron	0.87	0.57	0.30	Hyper	Up
FKBP1B	2	24274695	24274736	42	2	Intron	0.69	0.96	-0.28	Hypo	Up
GMPR	6	16260445	16260473	29	2	Intron	0.68	0.93	-0.26	Hypo	Up
HBEGF	5	139717277	139717324	48	2	Intron	0.76	0.47	0.29	Hyper	Down
IP6K3	6	33710284	33710320	37	2	Intron	0.27	0.60	-0.33	Hypo	Up
KALRN	3	124281329	124281373	45	2	Intron	0.77	0.51	0.26	Hyper	Down
KLHL29	2	23750256	23750281	26	2	Intron	0.88	0.52	0.36	Hyper	Up
LIFR	5	38601623	38601671	49	2	Upstream	0.60	0.86	-0.26	Hypo	Up
MGAT4A	2	99264172	99264216	45	2	Intron	0.23	0.53	-0.30	Hypo	Up
MTSS1	8	125727330	125727375	46	2	Intron	0.91	0.60	0.32	Hyper	Up
MYO7A	11	76905938	76908116	2179	3	Intron	0.62	0.97	-0.36	Hypo	Up
MYO7A	11	76898554	76898595	42	2	Intron	0.17	0.48	-0.30	Hypo	Up
NDRG1	8	134261878	134261928	51	3	Intron	0.04	0.46	-0.42	Hypo	Up
P2RY6	11	73000436	73000474	39	3	Intron	0.50	0.23	0.28	Hyper	Down
PGBD5	1	230554455	230554484	30	2	Intron	0.75	0.46	0.29	Hyper	Down
PHACTR3	20	58236433	58236475	43	3	Intron	0.37	0.66	-0.29	Hypo	Up
PHGDH	1	120267645	120267683	39	2	Intron	0.93	0.67	0.25	Hyper	Down
PRSS21	16	2869882	2869916	35	2	Intron	0.92	0.57	0.35	Hyper	Down
RGCC	13	42038266	42038305	40	2	Intron	0.11	0.42	-0.31	Hypo	Up/Down
RUNX2	6	45448204	45448233	30	2	Intron	0.83	0.56	0.28	Hyper	Up
SPATA13	13	24825973	24825999	27	4	Exon	0.06	0.31	-0.25	Hypo	Down
TIMP3	22	33197034	33197061	28	2	Exon	0.75	0.48	0.28	Hyper	Down
ZBTB16	11	114050079	114050114	36	2	Intron	0.24	0.54	-0.29	Hypo	Up

Source data are provided as a Source Data file. [a]The glucocorticoid treatment-associated DMRs (GC-DMRs) result from the DMR analysis of the WGBS data of CD4+ memory T-cells of a MS discordant MZ twin pair of which the MS-affected co-twin was very recently treated with GCs at the moment of blood collection (n=1). The GC-DMRs were identified using the DSS-single package[8], including a smoothing span of 100 bp, a minimum region length of 25 bp with ≥2 CpGs and a P-value<0.01. The absolute mean methylation difference had to be larger than 0.25, and to limit the number of false positives only GC-DMRs located in reported GC-response genes were considered. [b]Genomic coordinates are based on human genome build GRCh37/hg19.

Supplementary Table 10. Number of hyper- and hypomethylated CpGs in the EPIC array EWAS data of the 45 MZ twins clinically discordant for MS, according to different Δβ-values and P-value thresholds (n = 45 twin pairs).

Hypermethylated in MS-affected co-twin	#CpGs	%CpGs	Hypomethylated in MS-affected co-twin	#CpGs	%CpGs
Unadjusted for cell type composition			Unadjusted for cell type composition		
Mean Δβ-value>0	502222	59.1	Mean Δβ-values<0	347551	40.9
Mean Δβ-value>0.005	168434	62.2	Mean Δβ-value<-0.005	102184	37.8
Mean Δβ-value>0.01	50255	54.4	Mean Δβ-value<-0.01	42090	45.6
Mean Δβ-value>0 & P<0.001	2913	60.1	Mean Δβ-value<0 & P<0.001	1933	39.9
Mean Δβ-value>0.005 & P<0.001	2880	59.9	Mean Δβ-value<-0.005 & P<0.001	1930	40.1
Mean Δβ-value>0.01 & P<0.001	2496	58.1	Mean Δβ-value<-0.01 & P<0.001	1806	41.9
Adjusted for cell type composition			Adjusted for cell type composition		
Mean Δβ-value>0	475327	55.9	Mean Δβ-values<0	374446	44.1
Mean Δβ-value>0.005	101816	62.8	Mean Δβ-value<-0.005	60242	37.2
Mean Δβ-value>0.01	17129	63.8	Mean Δβ-value<-0.01	9712	36.2
Mean Δβ-value>0 & P<0.001	385	55.2	Mean Δβ-value<0 & P<0.001	313	44.8
Mean Δβ-value>0.005 & P<0.001	354	53.4	Mean Δβ-value<-0.005 & P<0.001	309	46.6
Mean Δβ-value>0.01 & P<0.001	249	53.9	Mean Δβ-value<-0.01 & P<0.001	213	46.1

Δβ-values = clinically MS-affected MZ co-twin – non-affected MZ co-twin. n= number of twin pairs, P = P-value two-tailed Wilcoxon signed-rank test.

Supplementary Table 11. Statistical power of the multiple sclerosis EWAS that includes 45 MZ twin pairs clinically discordant for multiple sclerosis.

Magnitude of the correlation	Power to detect a mean β-value difference of (at least) **0.05** at alpha = $1x10^{-7}$	Power to detect a mean β-value difference of (at least) **0.04** at alpha = $1x10^{-7}$	Power to detect a mean β-value difference of (at least) **0.03** at alpha = $1x10^{-7}$
0	0.984	0.749	0.210
0.2	0.999	0.914	0.390
0.4	>0.999	0.991	0.686
0.6	>0.999	>0.999	0.961
0.8	>0.999	>0.999	>0.999
1.0	>0.999	>0.999	>0.999

The table shows the statistical power to detect a mean β-value difference of (at least) 0.05, 0.04 and 0.03 with a (genome-wide) significance threshold of $1x10^{-7}$, a sample size of 45 MS discordant MZ twin pairs using a two-sided paired T-test and assuming a standard deviation of 0.0266 (which is the true median standard deviation observed in the EPIC array data).

Supplementary Table 12. Primer sequences and PCR conditions.[a]

Method	CpG/SNP number	Gene/ element	Forward primer sequence (5′→3′)	C	Reverse primer sequence (5′→3′)	C	T	Cyc	Product size	# CpGs
TDBS	cg12393503	*ECT2*	GATTTTGTGTGAGTGAGAGAGGTGT	133	TCTTCTATCCAAAAAAACCAACAAATA	133	58	42	252	19
	cg01447350	*IL34*	TTTTAGTTATTTGGGAGGTTGAAGTAG	133	ATCCATAAATAACTCAAACTAAAAAACAAA	133	59	42	340	8
	cg02520593	*SELPLG*	TTTGTTGTTTAAGAGGTAAAATTGAAGTT	133	ATATCCCAACTACAAATCCAATACAAA	133	58	42	230	3
	cg27037608	*TMEM232*	ATTAGGATTTATAAGTGAATTTTTATTTGTTT	133	CAAAACATTCTAAATACTTTATTTACTCACTA	133	60	40	379	9
	cg25345365	*ZBTB16*	ATTTTTTTGAGGGAAAGAATATATAGTGT	133	AATACAAAATATACCAAAACAACAAACC	133	60	42	191	3
		ALU[b]	TTTTAGTATTTTGGGAGGT	100	CCCAAACTAAAATACAATAAC	100	60	30	232	17
		HERVK[c]	TATTTTTTAATTTTAAGTATTTAGGGAT	100	TTCCTCTTATCTCAACTACAAAAA	100	56	30	233	6
		LINE1[d]	GGTTTATTTTATTAGGGAGTGTTAGAT	100	AAACCCTCTAAACCAAATATAAAATATAA	100	54	30	257	18
	BisConAssay	*PTPRVP*	TGGGGTAATGATGAGAGATGG	100	CTCTCTTTATTTCAAAACCCCCTA	100	58	40	343	NA
TDS	rs6471533		ACCCACTGGTTCTGGGAAG	133	TATGGCATGTTGGCAGAAGA	133	63	35	170	NA

[a]Loci were amplified in 30 µL mixes containing 40 ng bisulfite-treated DNA (TDS: 25 ng genomic DNA), 0.2 mM of each dNTP, *n* nM of each primer, 2.5 mM $MgCl_2$, 1.5 U HotStarTaq DNA polymerase (Qiagen) and 1X PCR buffer. DNA was denatured for 15 min at 95°C, followed by *n* cycles of 30 sec at 95°C, 1 min at T°C and 30 sec up to 1 min at 72°C. The reaction was completed by a final extension step of 5 min at 72°C. PCR products were mixed, purified using AMPure XP beads (Agencourt) and quantified using the Qubit Fluorometer and Qubit dsDNA HS assay kit (Invitrogen). *TMEM232* was sequenced with a minimum coverage of 1500 reads. All other amplicons were sequenced with a minimum coverage of at least 2000 reads. [b]Alu primers were designed using the consensus sequence published by Price et al.[9] (nucleotide positions 29-260) and generate *in silico*[10] an "infinite" number of specific PCR products (no mismatches allowed). [c]*HERVK* primers target the youngest subfamily LTR5Hs (nucleotide position 256-487) and generate *in silico*[10] 328 different specific PCR products (no mismatches allowed) of which 98% matches to LTR5Hs according to RepeatMasker (http://repeatmasker.org/). [d]*LINE1* Primers were designed using the promoter/5′-UTR consensus sequence (GenBank-Nr. X58075.1, nucleotide positions 105-361) and generate *in silico*[10] 309 different specific PCR products (no mismatches allowed), which mainly comprise the youngest subfamilies L1HS (~64%), L1PA2 (~25%) and L1PA3 (~9%). The repetitive elements were sequenced with a minimum coverage of 2000 reads, giving a >6 fold coverage per individual *HERVK* and *LINE1* element. BisConAssay = bisulfite conversion rate assay (non-bisulfite-dependent primers), C = primer concentration (nM), Cyc = number of cycles, NA = not applicable, T = annealing temperature (°C), TDBS = targeted deep bisulfite sequencing, TDS = targeted deep sequencing, #CpGs = number of CpGs present in the amplicon.

Supplementary Table 13. Sequencing coverage statistics of the whole genome bisulfite sequencing (WGBS) analysis in CD4+ memory T-cells of four MS discordant female MZ twin pairs.

Sample	Age	Years discordant for MS	SS	#CpGs	Average coverage	#CpGs with coverage ≥5	#CpGs with coverage ≥10	#CpGs with coverage ≥10 across all samples included in the MS-DMR analysis	Average coverage	#CpGs with coverage ≥15 across all samples included in the GC-DMR analysis	Average coverage
R-MS	39.8	14.4	N	27,951,337	11.0	25,375,958	15,495,007	2,693,926	19.1		
R-H	39.8	14.4	N	27,901,316	9.5	24,183,957	12,021,398	2,693,926	16.6		
AK-MS	46.5	19.1	Y	27,827,126	8.1	22,221,731	8,188,033	2,693,926	15.4		
AK-H	46.5	19.1	Y	27,962,853	11.4	25,245,516	16,062,214	2,693,926	20.4		
AV-MS	45.8	13.9	N	27,984,627	11.9	25,949,059	17,517,610	2,693,926	20.5		
AV-H	45.8	13.9	Y	27,972,888	10.9	25,398,997	15,269,579	2,693,926	19.1		
AY-MS[a]	40.9	12.0	Y	28,002,000	12.1	26,061,610	17,794,246	2,693,926	20.6	2,796,900	21.8
AY-H	40.9	12.0	N	27,970,390	10.8	25,398,195	15,270,455	2,693,926	18.7	2,796,900	20.6

[a]Only the MS-affected co-twin of pair AY had been treated very recently with GCs at the time of blood collection (but never received any immune-modulating therapy), while the MS-affected co-twins of the other three pairs had not received GCs or other immune-modulating therapies within at least 12 months prior to blood collection. GC-DMR = glucocorticoid treatment-associated differentially methylated region, MS-DMR = MS-associated DMR, SS = smoking status at sample collection (Y = yes, N = no).

Supplementary Figures

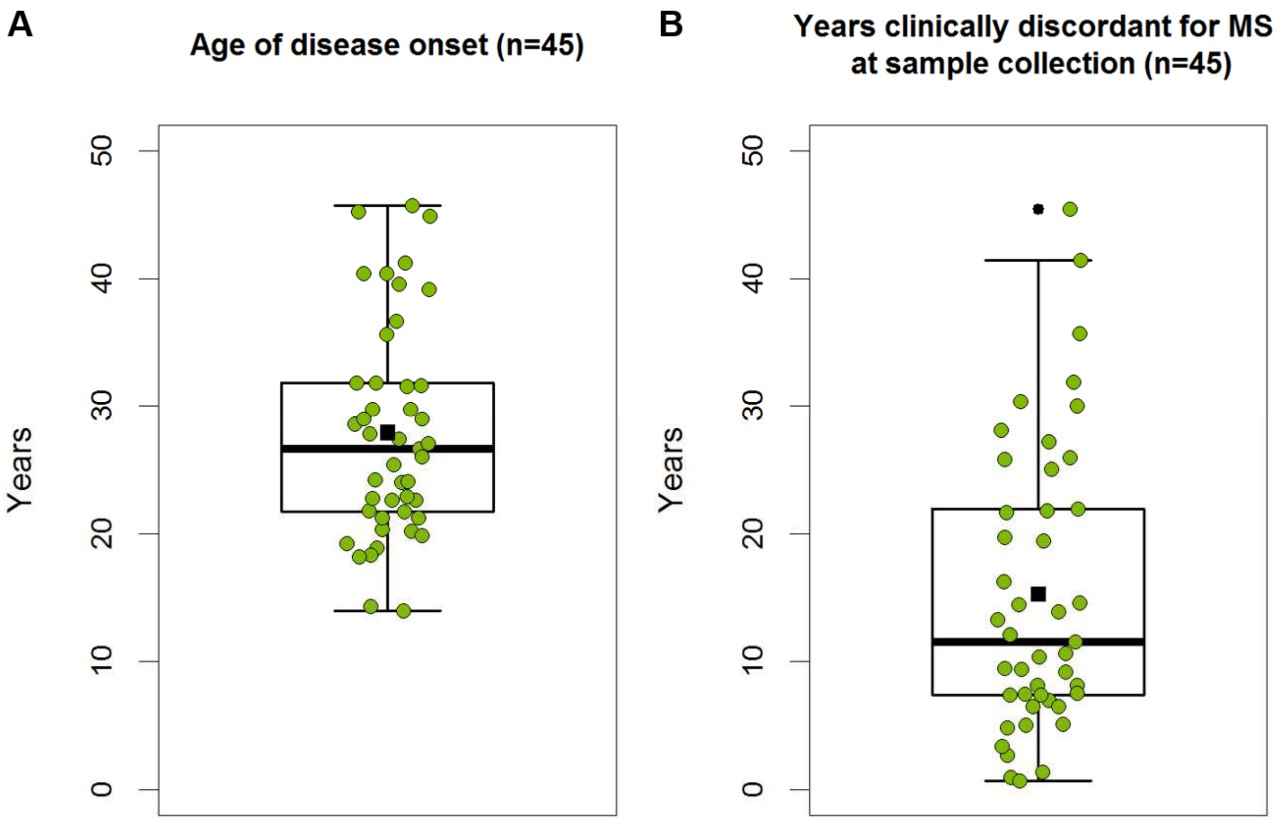

Supplementary Figure 1. Tukey boxplots (with all data points shown in green) of (A) the age of disease onset and (B) the years that the MZ twins were clinically discordant for MS at sample collection (n = 45 twin pairs). Our twin cohort has an average age of onset of 28 years, and contains 7 (16%) cases that were younger than 20 years and 6 (13%) cases that were older than 40 years at disease onset. Since MS has an average age of onset of about 30 years and manifests in 70% of the patients between 20 and 40 years of age[11,12], the age of onset in our cohort is within the normal range. Boxplots represent the median (central line), the interquartile range or IQR (bottom and top of the box), and 1.5 times the IQR (whiskers).

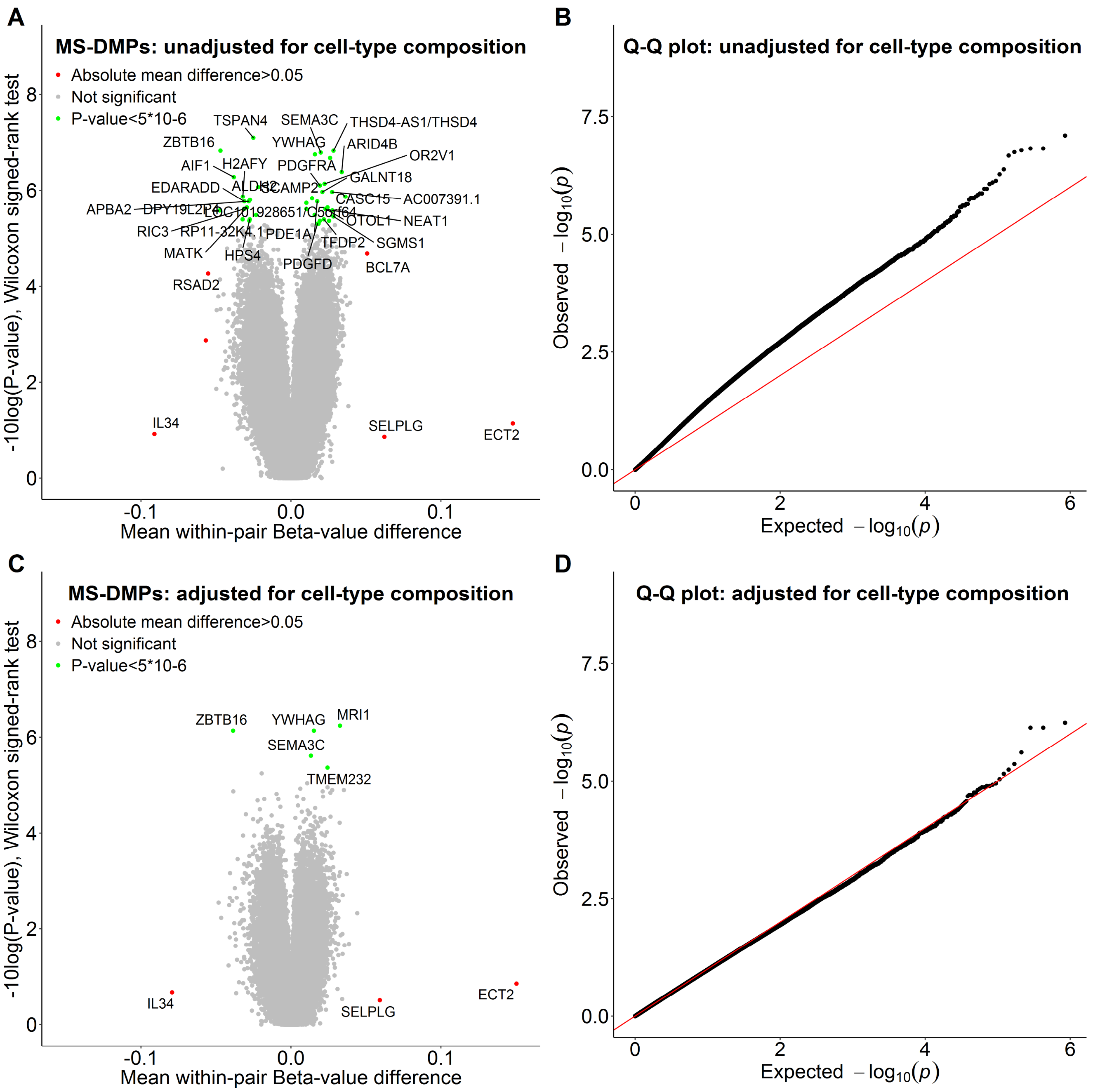

Supplementary Figure 2. DNA methylation changes associated with the clinical manifestation of MS (n = 45 twin pairs). Results of the differential DNA methylation analysis including the EPIC array data of all 45 MZ twin pairs clinically discordant for MS. **(A)** Volcano plot of the P-values resulting from the non-parametric two-tailed Wilcoxon signed-rank test versus the mean within-pair β-value difference for each CpG. Data were unadjusted for cell-type composition. **(B)** Q-Q plot of the P-values resulting from the non-parametric two-tailed Wilcoxon signed-rank shown in Figure 3A. Data were unadjusted for cell-type composition. **(C)** Volcano plot of the P-values resulting from the non-parametric two-tailed Wilcoxon signed-rank test against the mean within-pair β-value difference for each CpG. Data were adjusted for cell-type composition. **(D)** Q-Q plot of the P-values resulting from the non-parametric two-tailed Wilcoxon signed-rank shown in Figure 3C. Data were adjusted for cell-type composition. Within-pair β-value difference (Δβ-value) = clinically MS-affected MZ co-twin – non-affected MZ co-twin.

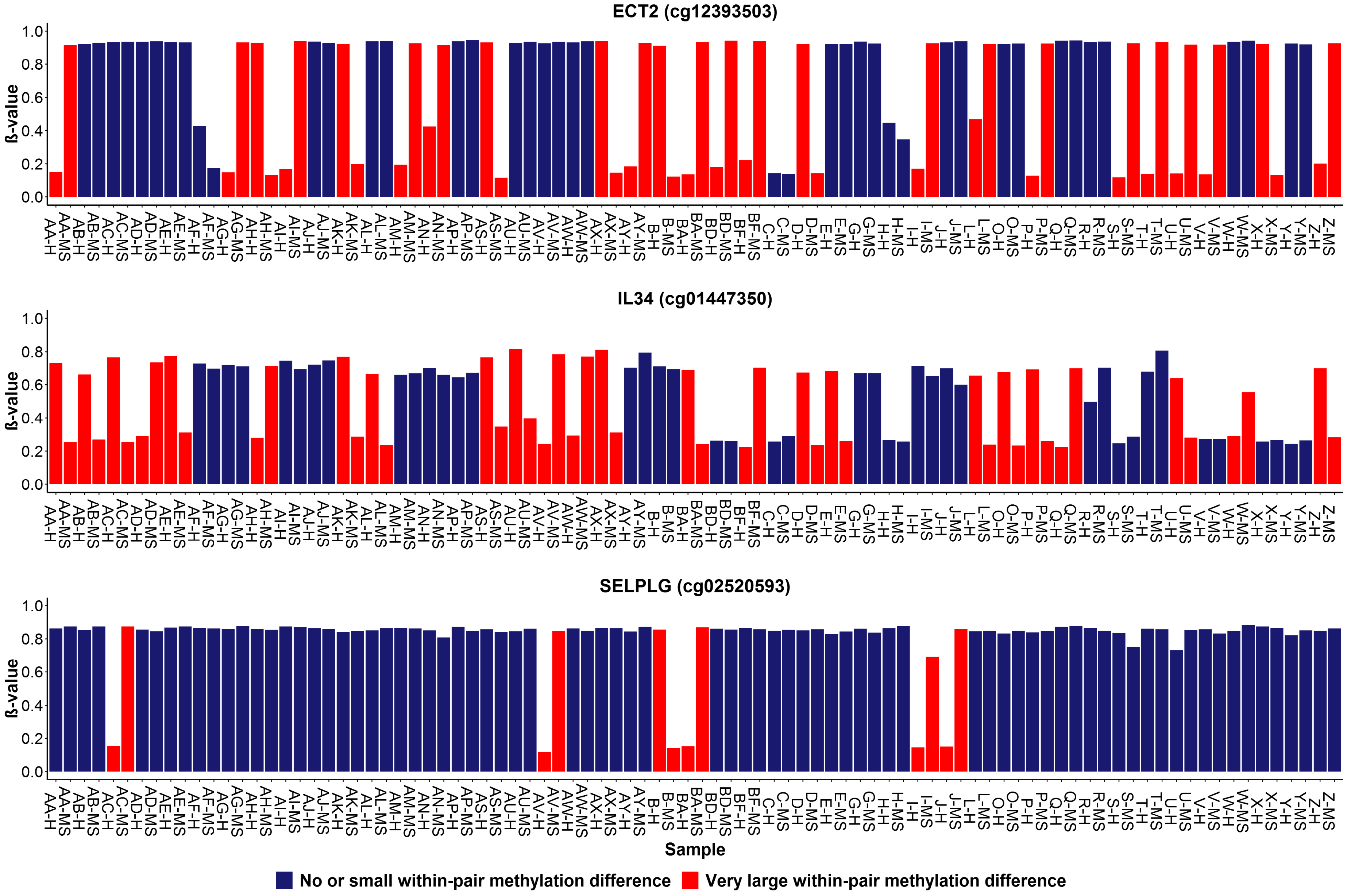

Supplementary Figure 3. Infinium MethylationEPIC BeadChip β-values of each sample are shown for the *ECT2* (cg12393503), *SELPLG* (cg02520593) and *IL34* (cg01447350) CpGs, which show very large mean within-pair β–value differences (n = 45 twin pairs). Labels indicate: Pair ID - Disease status (i.e. MS = MS-affected MZ co-twin, H = clinically non-affected MZ co-twin).

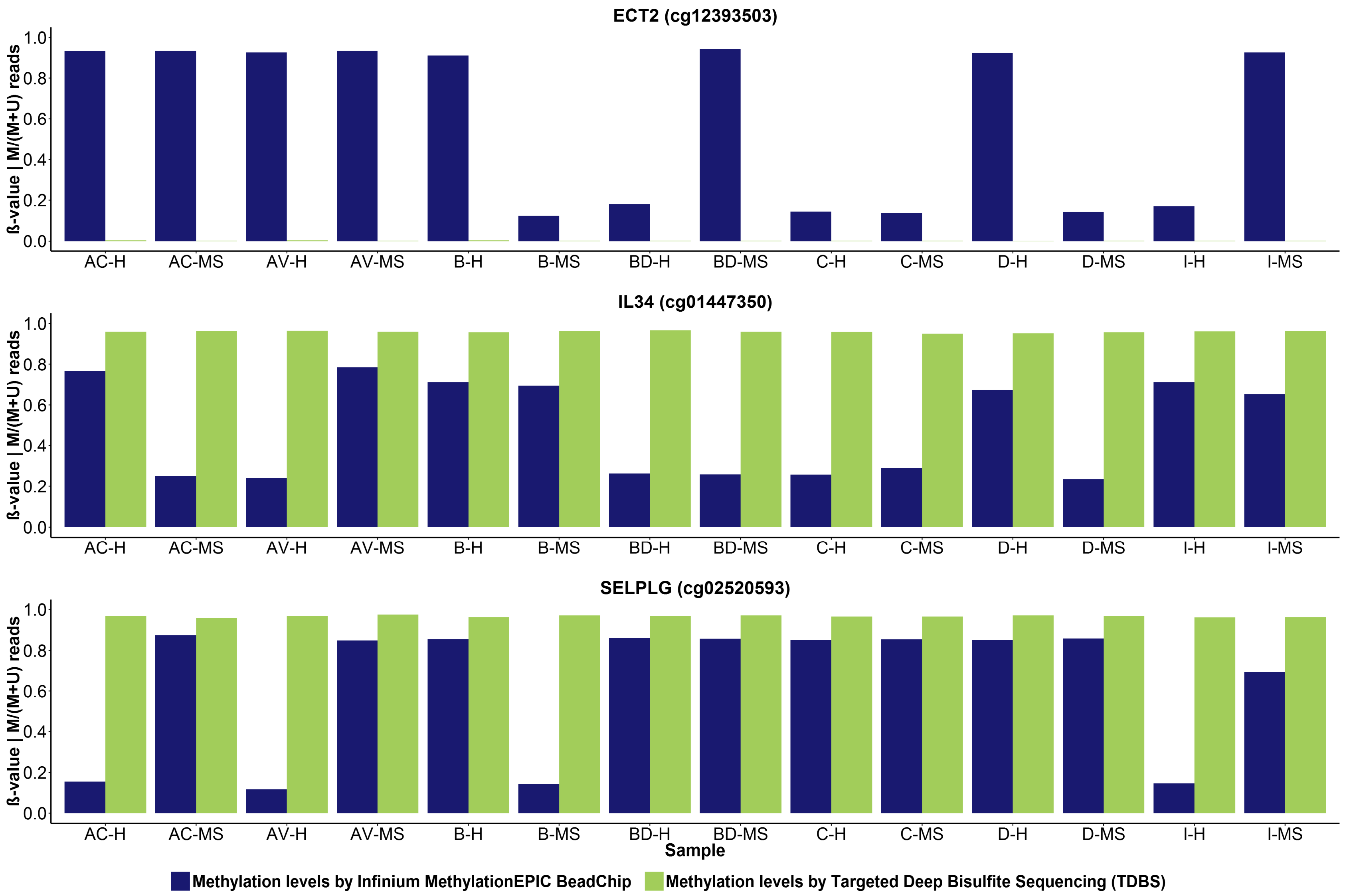

Supplementary Figure 4. Validation of the *ECT2* (cg12393503), *IL34* (cg01447350) and the *SELPLG* (cg02520593) CpGs by targeted deep bisulfite sequencing (TDBS) (n = 7 twin pairs). On the y-axis the Infinium MethylationEPIC BeadChip β-values as well as the TDBS results are shown, both represented as the fraction of methylated cytosines. Labels indicate: Pair ID - Disease status (i.e. MS = MS-affected MZ co-twin, H = clinically non-affected MZ co-twin), M = methylated, U = unmethylated. In contrast to the Infinium MethylationEPIC BeadChip data, TDBS revealed that *ECT2* (cg12393503) was completely unmethylated, while *IL34* (cg01447350) and *SELPLG* (cg02520593) were highly methylated in all samples.

A

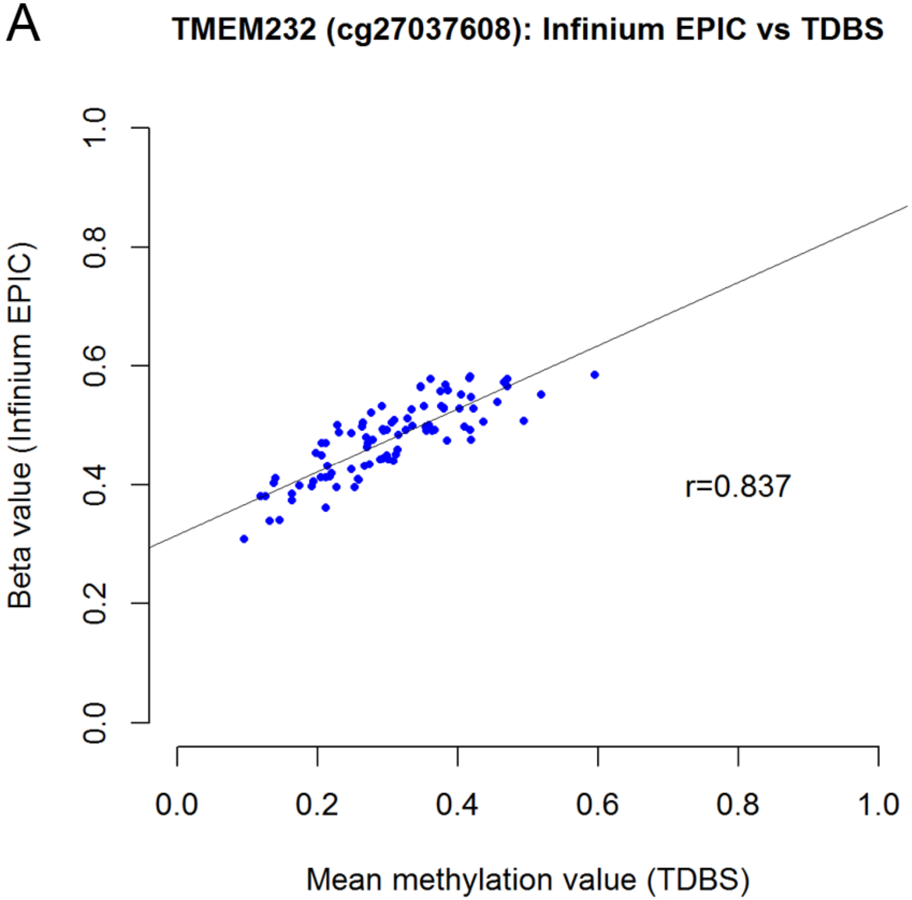

B

n = 45		Mean methylation				
Method	*TMEM232*	MS-affected co-twin	non-affected co-twin	Mean within-pair methylation difference (U) (95% CI)	P_{W-U}	Methylation range
**EPIC**	**cg27037608**	**0.488**	**0.466**	**0.022 (0.012,0.032)**	**4.8*10^{-5}**	**0.31-0.59**
**TDBS**	CpG-1	0.223	0.206	0.017 (-0.002,0.036)	0.11	0.05-0.42
	CpG-2	0.250	0.226	0.024 (0.006,0.042)	0.01	0.05-0.47
	CpG-3	0.288	0.258	0.030 (0.010,0.050)	7.4*10^{-3}	0.06-0.57
	CpG-4	0.285	0.256	0.029 (0.009,0.049)	4.9*10^{-3}	0.07-0.55
	**cg27037608**	0.315	0.292	**0.023 (0.002,0.043)**	**0.05**	**0.09-0.60**
	CpG-6	0.275	0.247	0.028 (0.008,0.048)	0.02	0.07-0.49
	CpG-7	0.273	0.240	0.035 (0.015,0.055)	5.4*10^{-4}	0.06-0.53
	CpG-8	0.293	0.260	0.033 (0.014,0.051)	4.1*10^{-4}	0.07-0.57
	CpG-9	0.269	0.248	0.021 (0.002,0.040)	0.06	0.08-0.47
	Amplicon	0.274	0.248	0.026 (0.008,0.045)	7.4*10^{-3}	0.07-0.52

TMEM232 amplicon contains nine CpGs of which cg27037608 is the fifth and is located at chr5:110,062,618. Genomic coordinates are based on human genome build GRCh37/ hg19. CI = confidence interval (parametric), n = number of MS-discordant twin pairs, TDBS = targeted deep bisulfite sequencing, P_{W-U} = P-value nonparametric two-tailed Wilcoxon signed-rank test unadjusted for cell-type composition, U = unadjusted for cell-type composition.

Supplementary Figure 5. Validation of the *TMEM232* (cg27037608) MS-DMP by targeted deep bisulfite sequencing (TDBS) (n = 45 twin pairs). (A) Correlation plot of the unadjusted Infinium MethylationEPIC BeadChip data and the TDBS data of the *TMEM232* (cg27037608) MS-DMP of all 45 MZ twin pairs. Infinium MethylationEPIC BeadChip data are expressed as β-value. TDBS data are expressed as mean methylation value, where the methylation level is calculated by dividing the number of reads in which the particular CpG is methylated by the total number of sequenced reads (minimal coverage >1500 reads/base). r = Pearson's correlation coefficient with P-value. **(B)** Summary of the non-parametric two-tailed Wilcoxon signed-rank test on the unadjusted Infinium MethylationEPIC data and the TDBS data of the *TMEM232* amplicon including the cg27037608 DMP (n = all 45 twin pairs). Within-pair methylation difference = MS-affected MZ co-twin – clinically non-affected MZ co-twin. Source data are provided as a Source Data file.

A

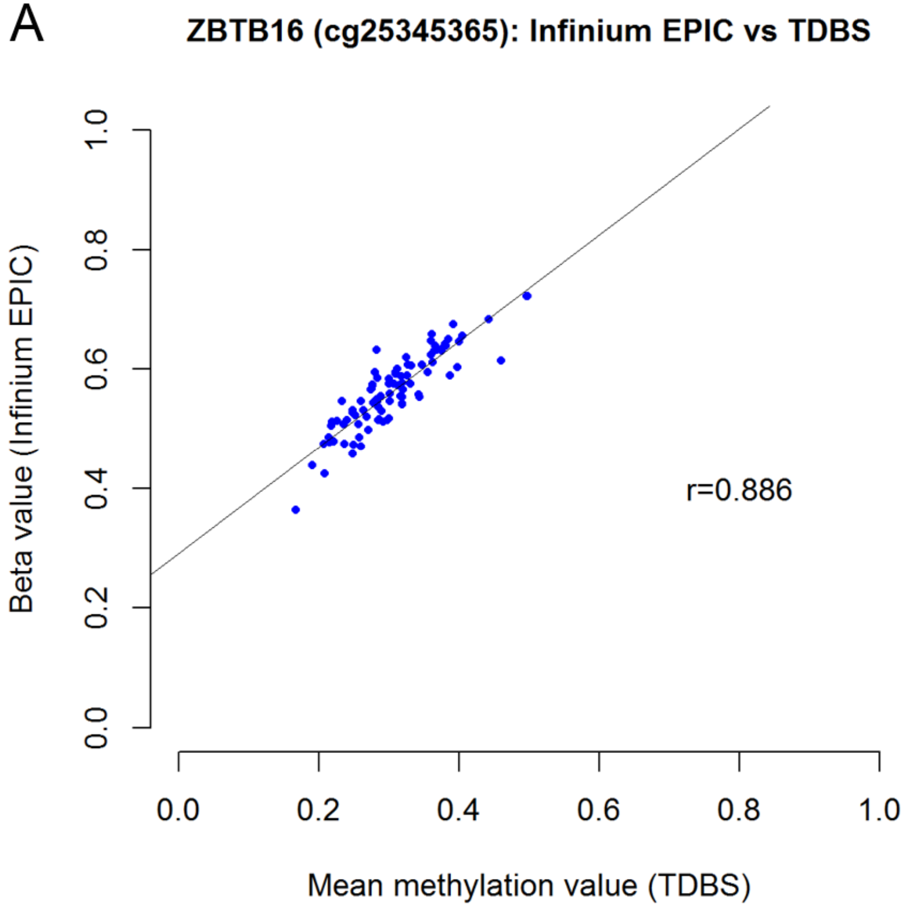

B

n = 45		Mean methylation				
Method	*ZBTB16*[a]	MS-affected co-twin	non-affected co-twin	Mean within-pair methylation difference (U) (95% CI)	$P_{W\text{-}U}$	Methylation range
**EPIC**	**cg25345365**	**0.540**	**0.587**	**-0.047 (-0.063,-0.031)**	**$1.5*10^{-7}$**	**0.36-0.72**
**TDBS**	CpG-1	0.557	0.604	-0.047 (-0.062,-0.032)	$3.8*10^{-7}$	0.37-0.75
	**cg25345365**	0.281	0.330	**-0.049 (-0.068,-0.030)**	**$1.4*10^{-6}$**	**0.17-0.50**
	CpG-3	0.170	0.195	-0.025 (-0.039,-0.011)	$1.2*10^{-3}$	0.07-0.32
	Amplicon	0.336	0.377	-0.040 (-0.055,-0.026)	$5.3*10^{-7}$	0.26-0.51

[a] *ZBTB16* amplicon contains three CpGs of which cg25345365 is the second and is located at chr11:114,050,114. CpG-1 is located at chr11:114,050,079 and CpG-3 is located at chr11:114,050,174. Genomic coordinates are based on human genome build GRCh37/ hg19. CI = confidence interval, n = number of MS-discordant MZ twin pairs, TDBS = targeted deep bisulfite sequencing, $P_{W\text{-}U}$ = P-value two-tailed Wilcoxon signed-rank test unadjusted for cell-type composition, U = unadjusted for cell-type composition.

Supplementary Figure 6. Validation of the *ZBTB16* (cg25345365) MS-DMP by targeted deep bisulfite sequencing (TDBS) (n = 45 twin pairs). (A) Correlation plot of the unadjusted Infinium MethylationEPIC BeadChip data and the TDBS data of the *ZBTB16* (cg25345365) MS-DMP of all 45 MZ twin pairs. Infinium MethylationEPIC BeadChip data are expressed as β-value. TDBS data are expressed as mean methylation value, where the methylation level is calculated by dividing the number of reads in which the particular CpG is methylated by the total number of sequenced reads (minimal coverage >2000 reads/base). r = Pearson's correlation coefficient with P-value. **(B)** Summary of the nonparametric two-tailed Wilcoxon signed-rank test on the unadjusted Infinium MethylationEPIC BeadChip data and the TDBS data of the *ZBTB16* (cg25345365) MS-DMP (n = 45 twin pairs). Within-pair methylation difference = MS-affected MZ co-twin – clinically non-affected MZ co-twin. Source data are provided as a Source Data file.

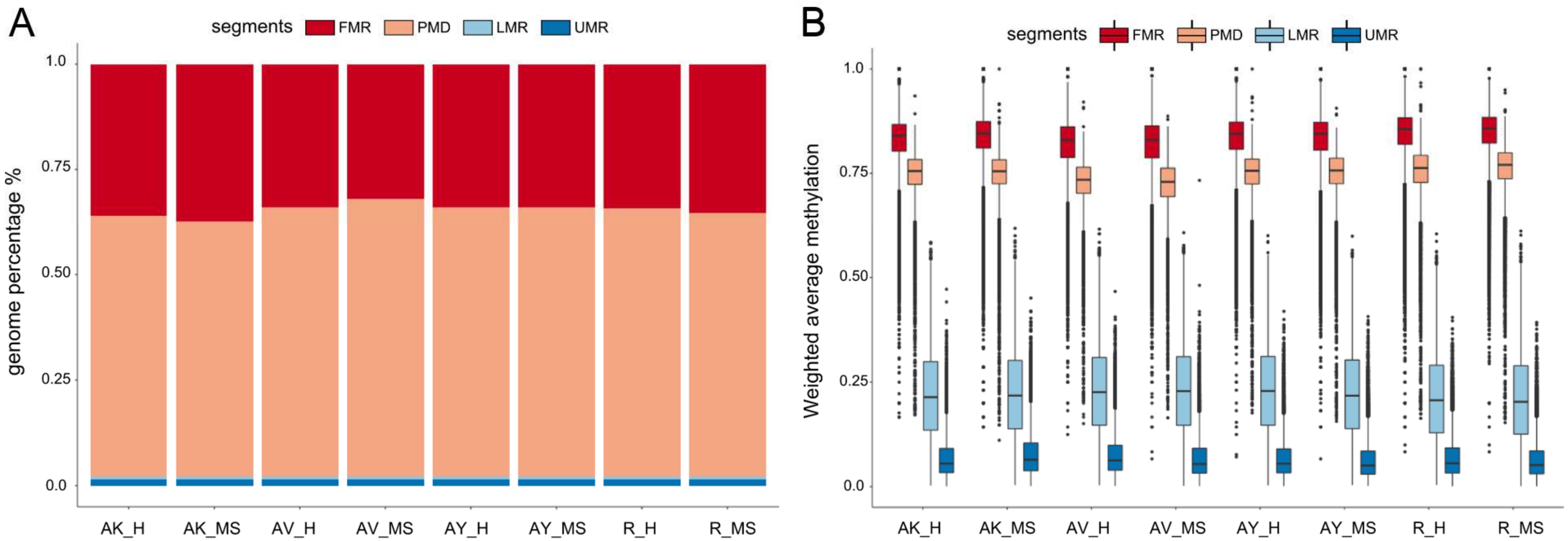

Supplementary Figure 7. Results of the PMD analysis on the WGBS data of CD4+ memory T-cells of four MS discordant MZ twin pairs (n = 4 twin pairs). Median weighted average methylation levels in PMDs, FMRs, LMRs and UMRs were similar between the clinically non-affected and MS-affected MZ co-twins (P>0.05, two-tailed paired T-test) **(A)** Segment percentages in the genome plotted as stacked barplots. **(B)** Weighted average methylation levels per segment plotted as boxplots. FMR = fully methylated region, PMD = partially methylated domains, LMR = low methylated region, UMR = unmethylated region. Labels indicate: Pair ID - Disease status (i.e. MS = MS-affected MZ co-twin, H = clinically non-affected MZ co-twin). The MS-affected co-twin of pair AY was treated with glucocorticoid at the moment of blood collection.

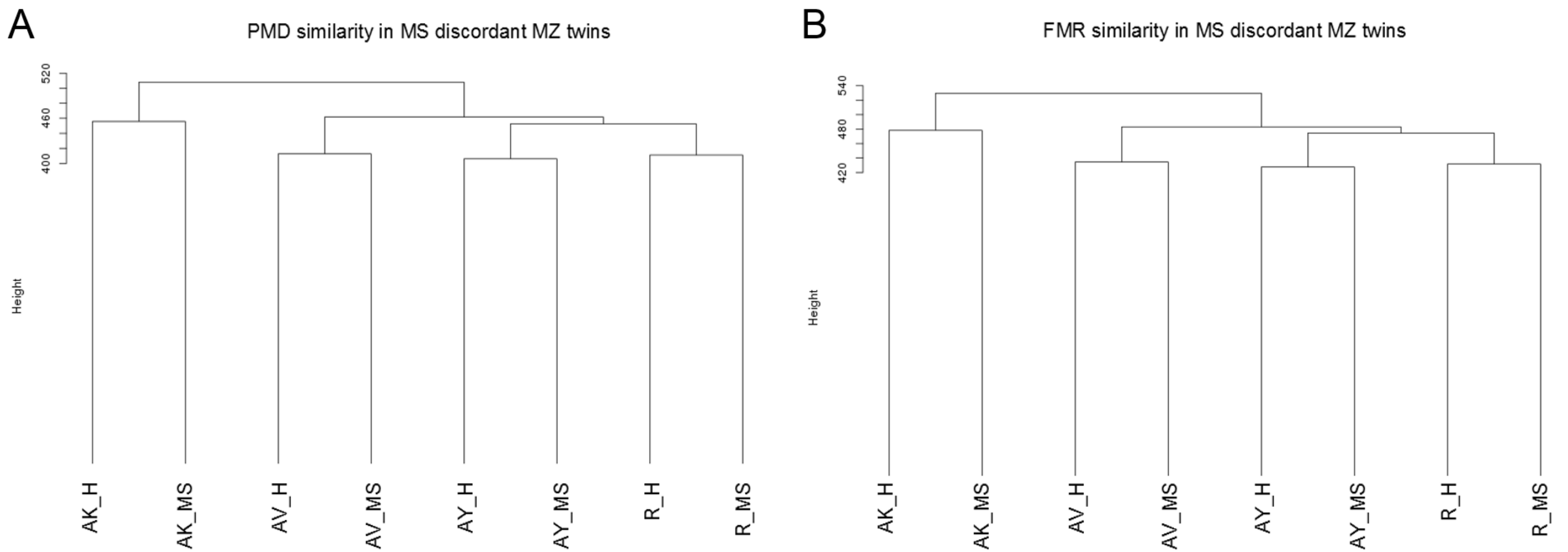

Supplementary Figure 8. Hierarchical clustering of (A) the partially methylated domains (PMDs) and (B) the fully methylated regions (FMRs), identified in the WGBS data of CD4+ memory T-cells of four MS discordant MZ twin pairs, demonstrating that all co-twins cluster together (n = 4 twin pairs). Labels indicate: Pair ID - Disease status (i.e. MS = MS-affected MZ co-twin, H = clinically non-affected MZ co-twin). The MS-affected co-twin of pair AY was treated with glucocorticoid at the moment of blood collection.

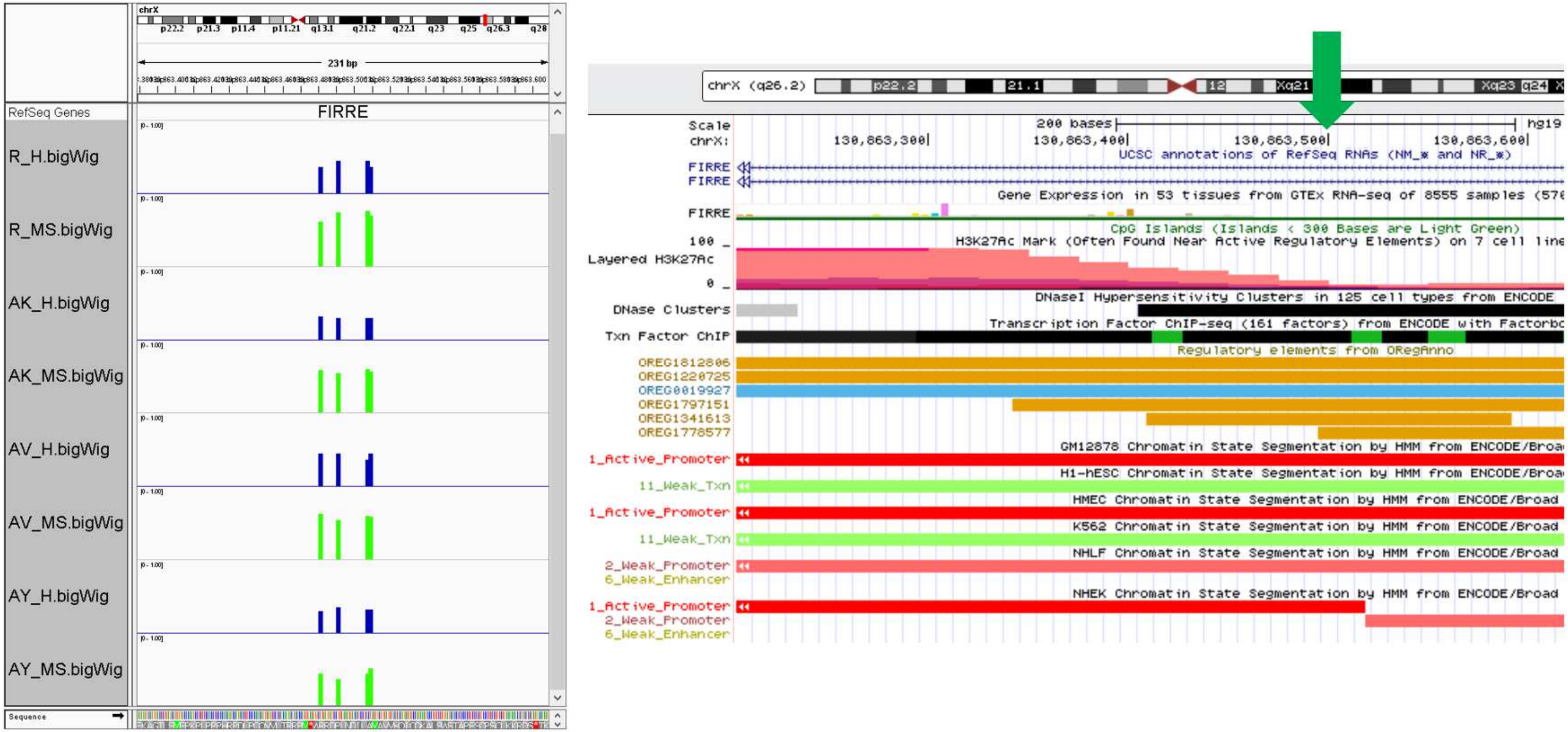

Chr	Start	End	Annotation	*Gene*	Mean methylation MS co-twins	Mean methylation unaffected co-twins	Mean within-pair methylation difference	Full name
X	130863481	130863509	Intron	*FIRRE*	0.66	0.40	0.26	Functional intergenic repeating RNA element

Supplementary Figure 9. MS-DMR in the *FIRRE* gene identified by WGBS of CD4+ memory T-cells of four female MS discordant MZ twin pairs (n = 4 twin pairs). This MS-DMR is located in an intronic CTCF/YY1 bound regulatory region in the *FIRRE* gene,[13] that is located on the X-chromosome (chrX:130863481-130863509) and encodes a circular long non-coding RNA.[14] MS-DMRs were defined as ≥3 CpGs, each having P-value<0.05 (two-tailed paired T test) and absolute mean methylation difference >0.2, and a maximum 500 bp distance between neighbouring CpGs. The green bars highlights the MS-affected co-twins. All genome coordinates are based on human genome build GRCh37/hg19. Labels indicate: Pair ID - Disease status (i.e. MS = MS-affected MZ co-twin, H = clinically non-affected MZ co-twin). Source data are provided as a Source Data file.

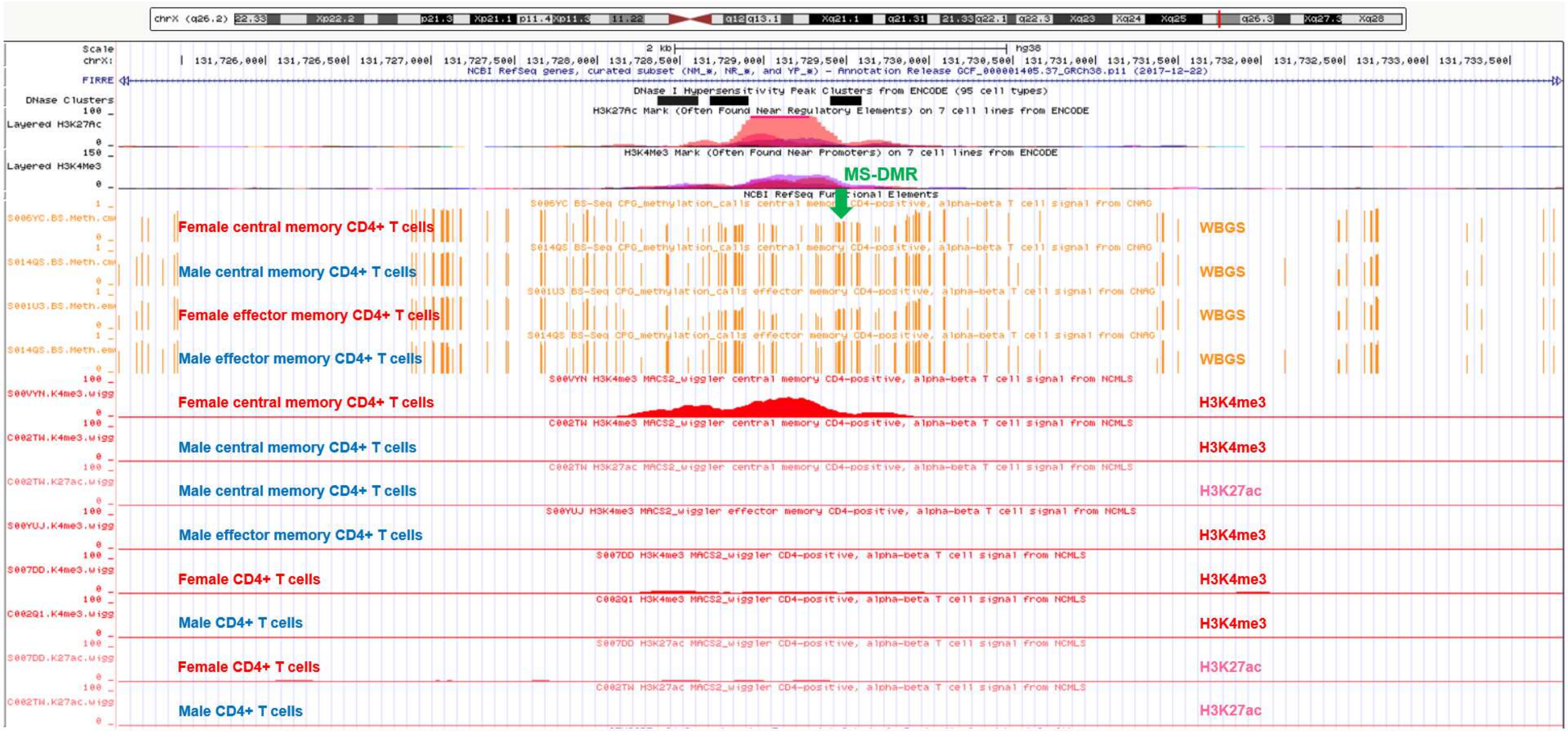

Supplementary Figure 10. **Methylation and chromatin status of the *FIRRE* MS-DMR in various subsets of primary CD4+ T cells in male and female BLUEPRINT samples[15].** Whole genome bisulfite sequencing (WGBS) data of central memory and effector memory CD4+ T cells shows that in females methylation levels at the *FIRRE* DMR locus are lower compared to males (~50% versus ~100%). In addition, in female central memory CD4+ T cells a H3K4me3 peak is observed at the *FIRRE* DMR locus, but not in males. Unfortunately, H3K27ac central memory and H3K4me3 effector memory CD4+ T-cell data was not available of a female donor. All genome coordinates are based on human genome build GRCh38/hg38.

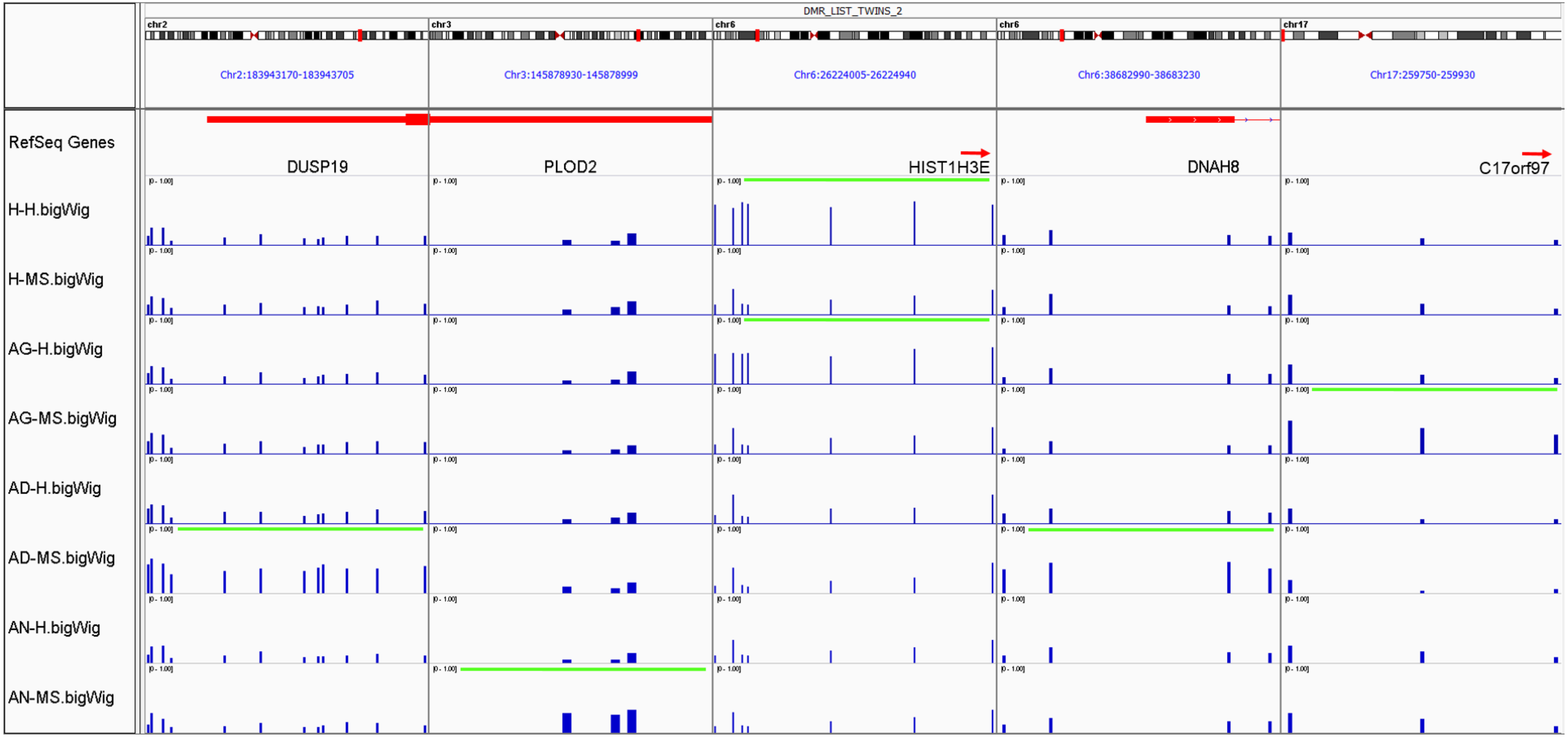

Supplementary Figure 11. Identified within-pair DMRs (WP-DMRs) in *DUSP19*, *PLOD2*, *HIST1H3E*, *DNAH8* and C17orf97. The green horizontal line highlights the aberrant methylated sample(s). These WP-DMRs were identified in the Infinium MethylationEPIC BeadChip data. Labels indicate: Pair ID - Disease status (i.e. MS = MS-affected MZ co-twin, H = clinically non-affected MZ co-twin). Source data are provided as a Source Data file.

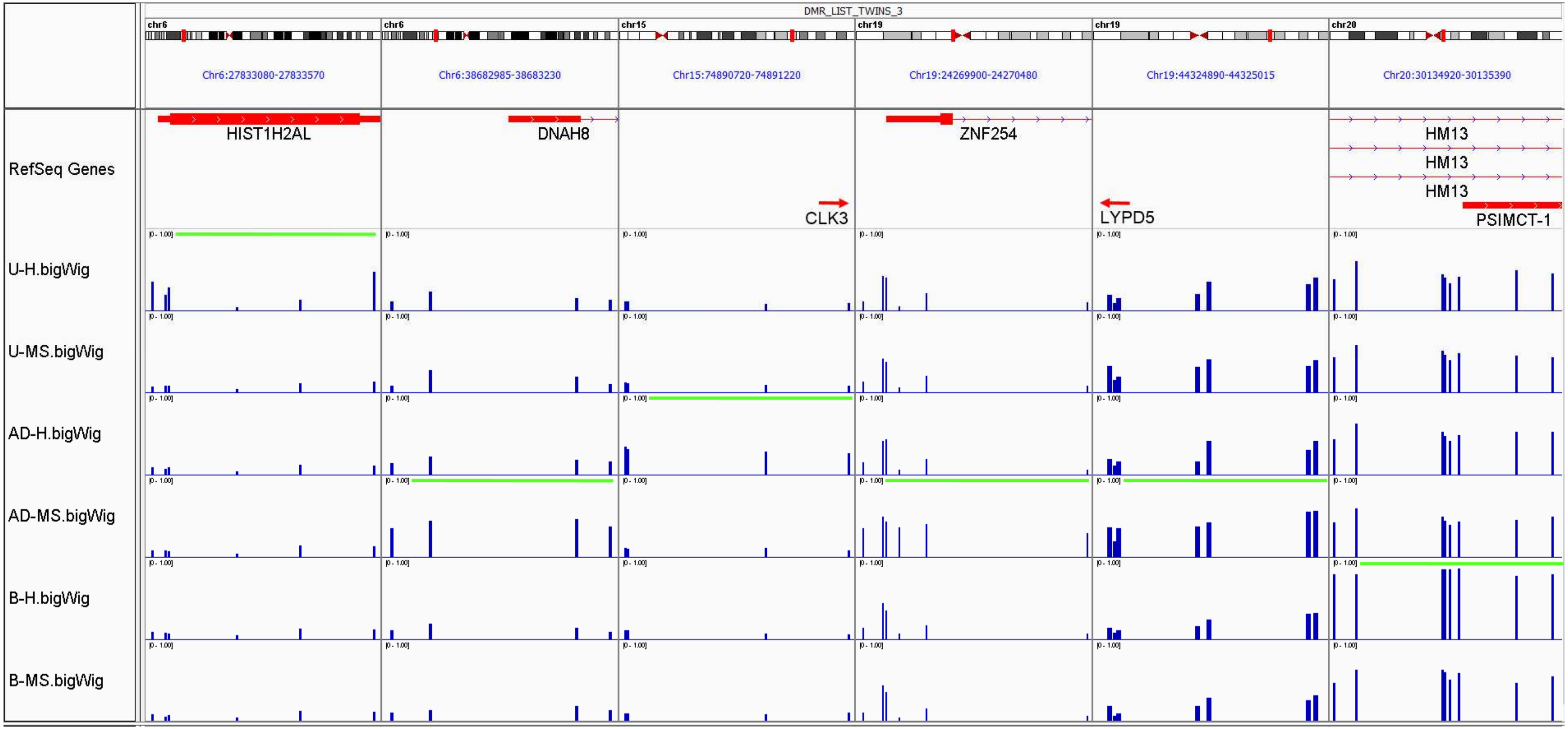

Supplementary Figure 12. Identified within-pair DMRs (WP-DMRs) in *HIST1H2AL, DNAH8, CLK3, ZNF254, LYPD5* and *HM13/MCTS2P*. The green horizontal line highlights the aberrant methylated sample. These WP-DMRs were identified in the Infinium MethylationEPIC BeadChip data. Labels indicate: Pair ID - Disease status (i.e. MS = MS-affected MZ co-twin, H = clinically non-affected MZ co-twin). *MCTS2P* is also called *PSIMCT-1*. Source data are provided as a Source Data file.

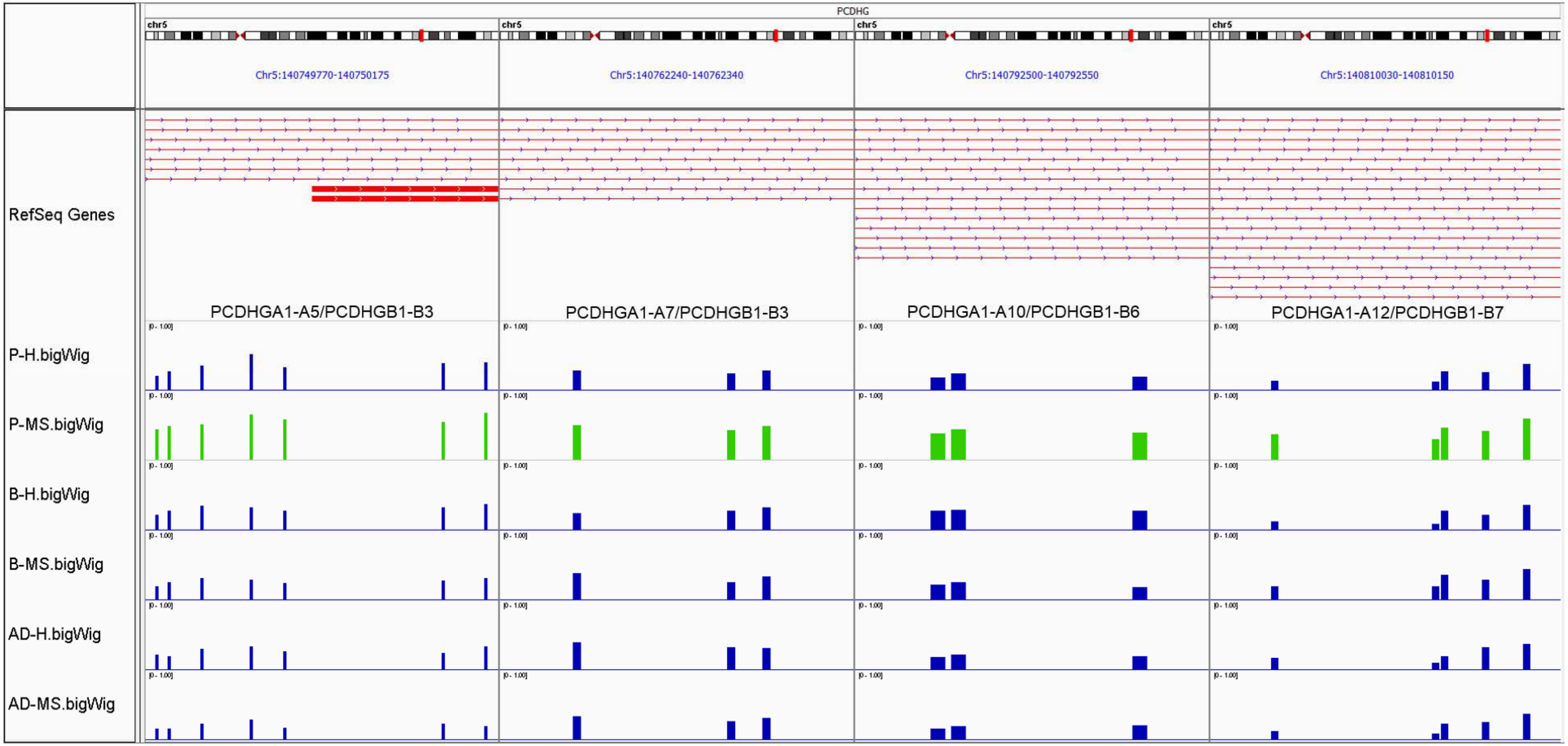

Supplementary Figure 13. Identified within-pair DMRs (WP-DMRs) in the *PCDHG* gene cluster. The green bars highlights the aberrant methylated sample. These WP-DMRs were identified in the Infinium MethylationEPIC BeadChip data. Labels indicate: Pair ID - Disease status (i.e. MS = MS-affected MZ co-twin, H = clinically non-affected MZ co-twin). Source data are provided as a Source Data file.

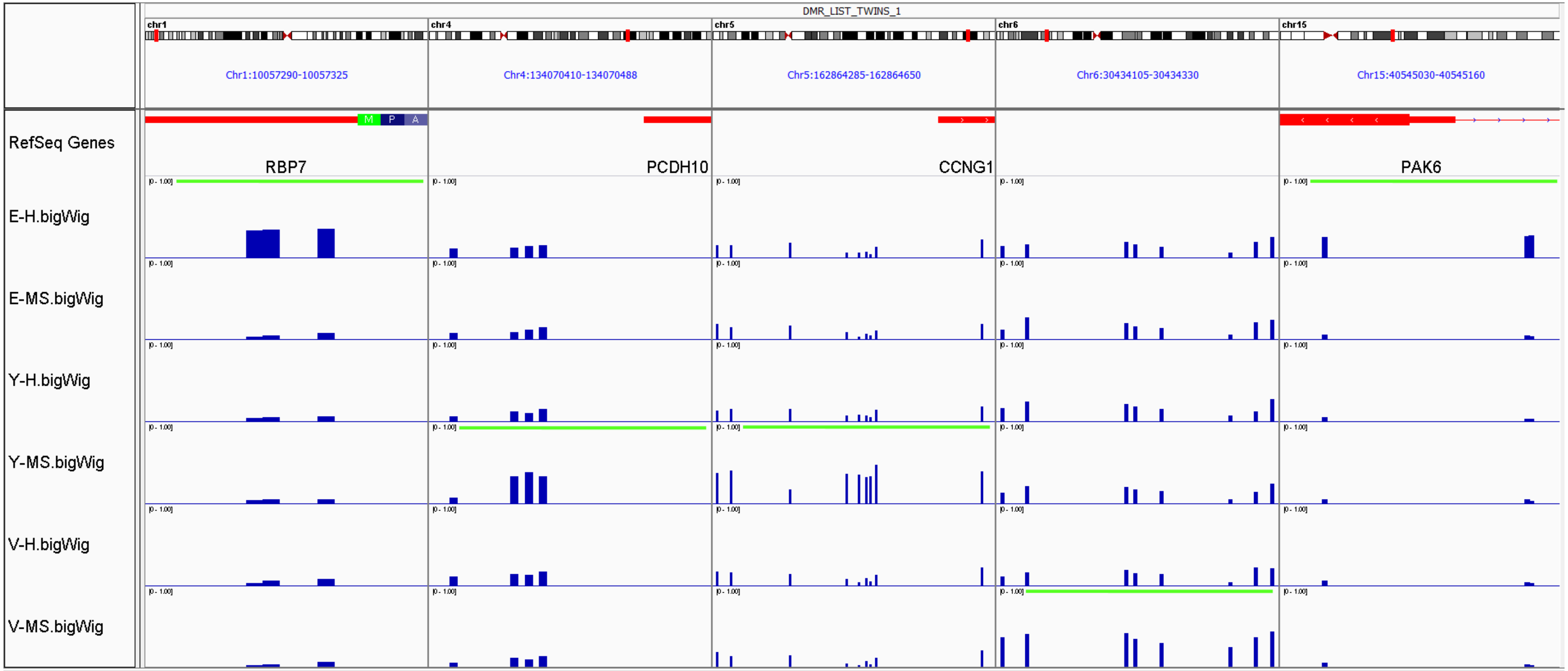

Supplementary Figure 14. Identified within-pair DMRs (WP-DMRs) in *RBP7*, *PCDH10*, *CCNG1*, Chr6:30434109-30434324 and *PAK6*. The green horizontal line highlights the aberrant methylated sample. These WP-DMRs were identified in the Infinium MethylationEPIC BeadChip data. Labels indicate: Pair ID - Disease status (i.e. MS = MS-affected MZ co-twin, H = clinically non-affected MZ co-twin). Source data are provided as a Source Data file.

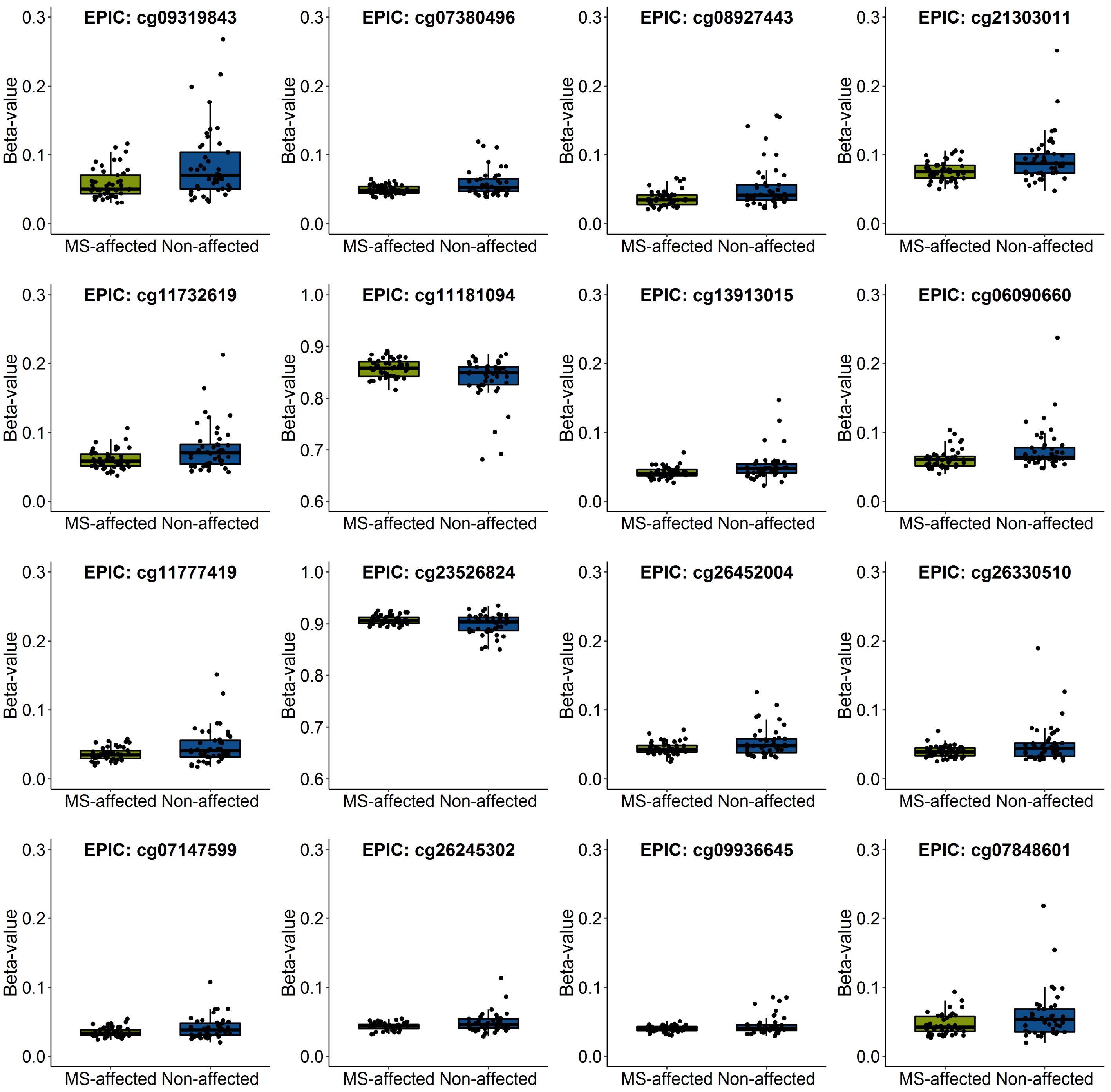

Supplementary Figure 15. Boxplots of the 16 top-ranked differentially variable positions (DVPs) identified in the 45 MS-discordant MZ twin pairs (n=45 twin pairs). DVPs were identified using the iEVORA algorithm[5] and were defined as CpGs with a FDR-corrected Barlett's P-value<0.001 and raw T-test P-value<0.05. Boxplots represent the median (central line), the interquartile range or IQR (bottom and top of the box), and 1.5 times the IQR (whiskers). Source data are provided as a Source Data file.

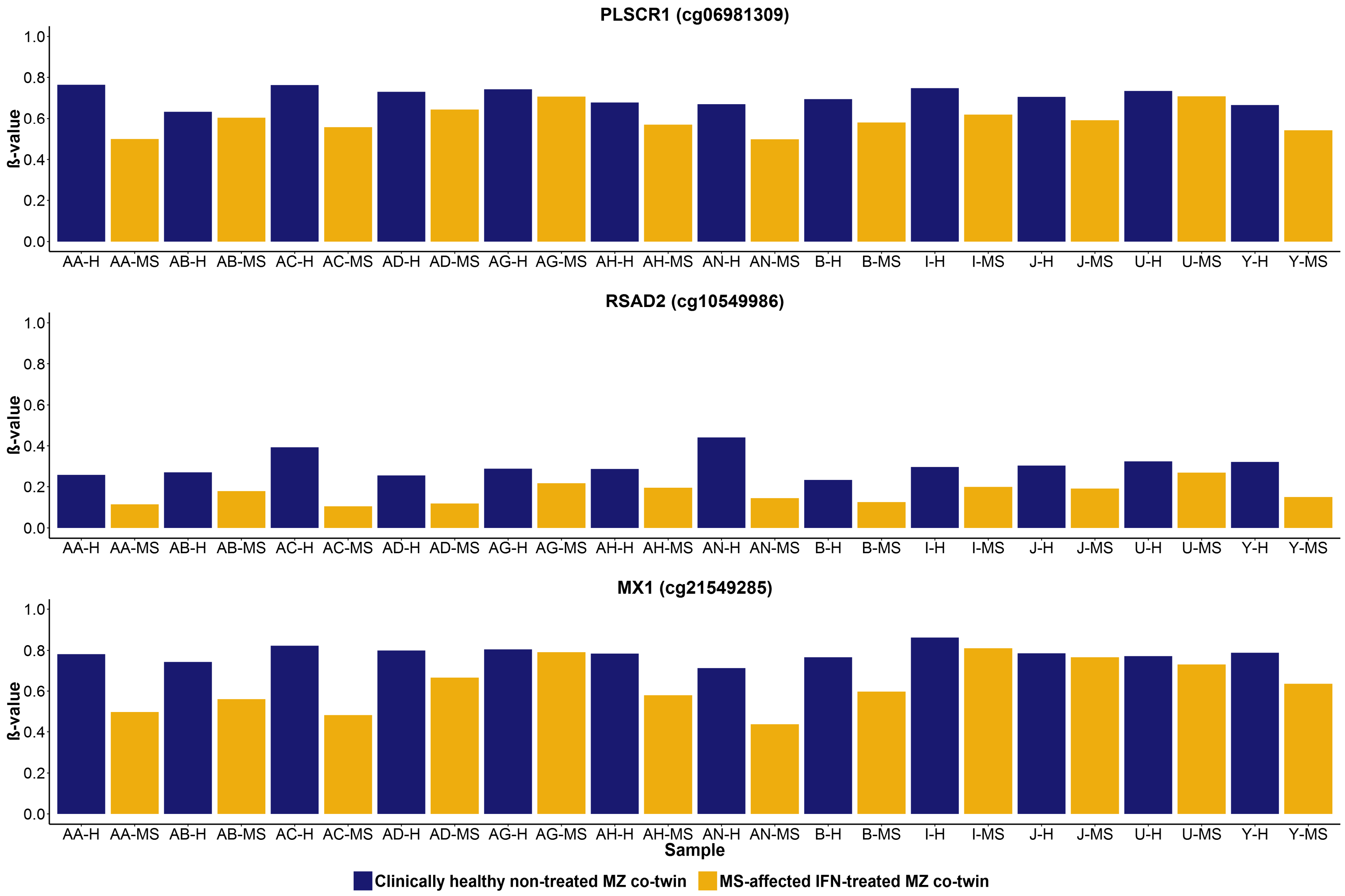

Supplementary Figure 16. Infinium MethylationEPIC BeadChip β-values of CpGs in *PLSCR1* (cg06981309), *RSAD2* (cg10549986) and *MX1* (cg21549285) that were strongly differentially methylated following interferon-beta (IFN) treatment (IFN-DMPs) (n = 12 twin pairs). Labels indicate: Pair ID - Disease status (i.e. MS = MS-affected IFN-treated MZ co-twin (yellow), H = clinically non-affected non-treated MZ co-twin (blue)). Source data are provided as a Source Data file.

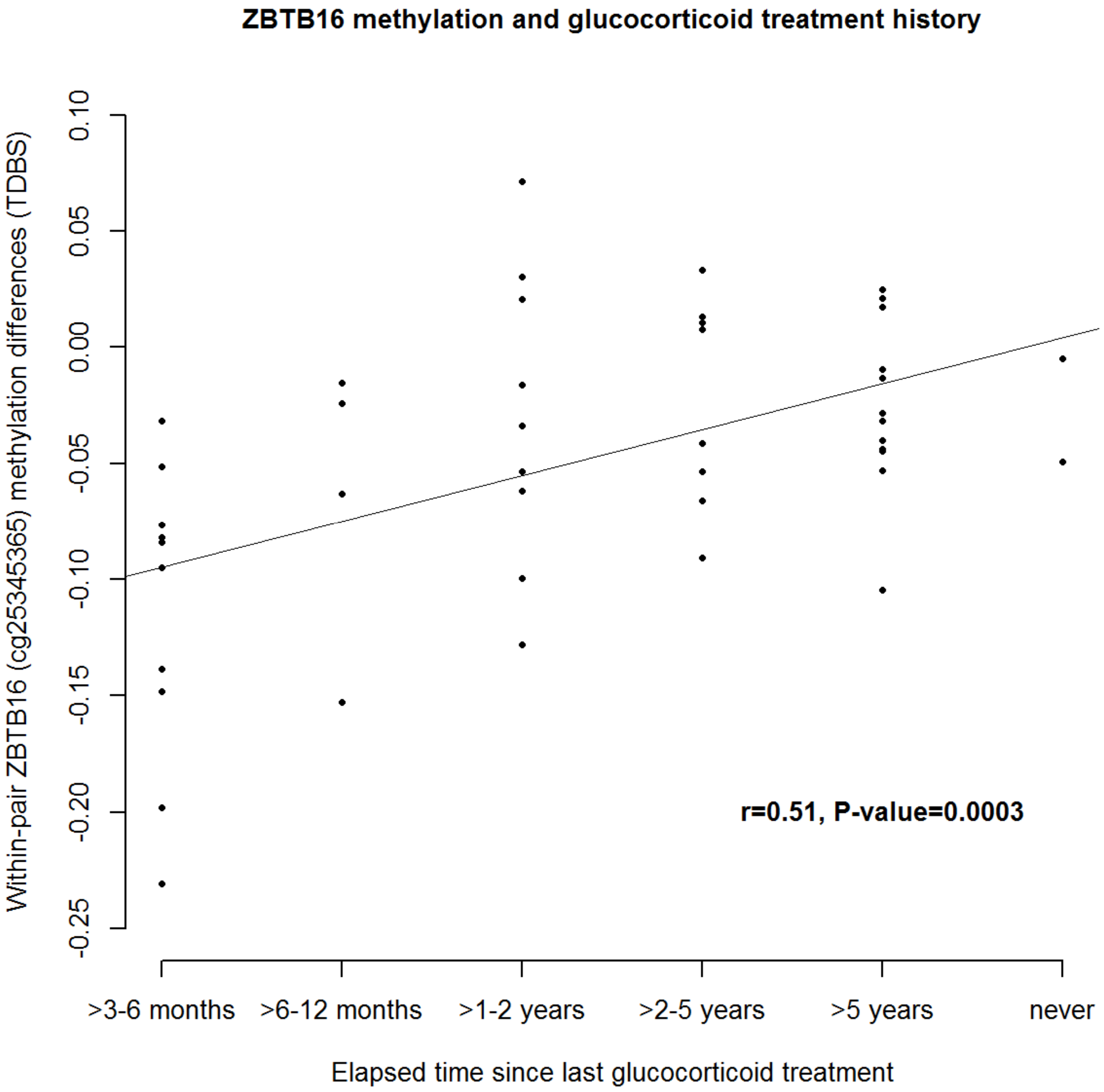

Supplementary Figure 17. *ZBTB16* MS-DMP (cg25345365, targeted deep bisulfite sequencing (TDBS)) within-pair methylation differences plotted against the elapsed time since the last glucocorticoid treatment in the MS-affected MZ co-twin (n = 45 twin pairs). r = Pearson's correlation coefficient with P-value, within-pair methylation difference = MS-affected MZ co-twin – clinically non-affected MZ co-twin. Source data are provided as a Source Data file.

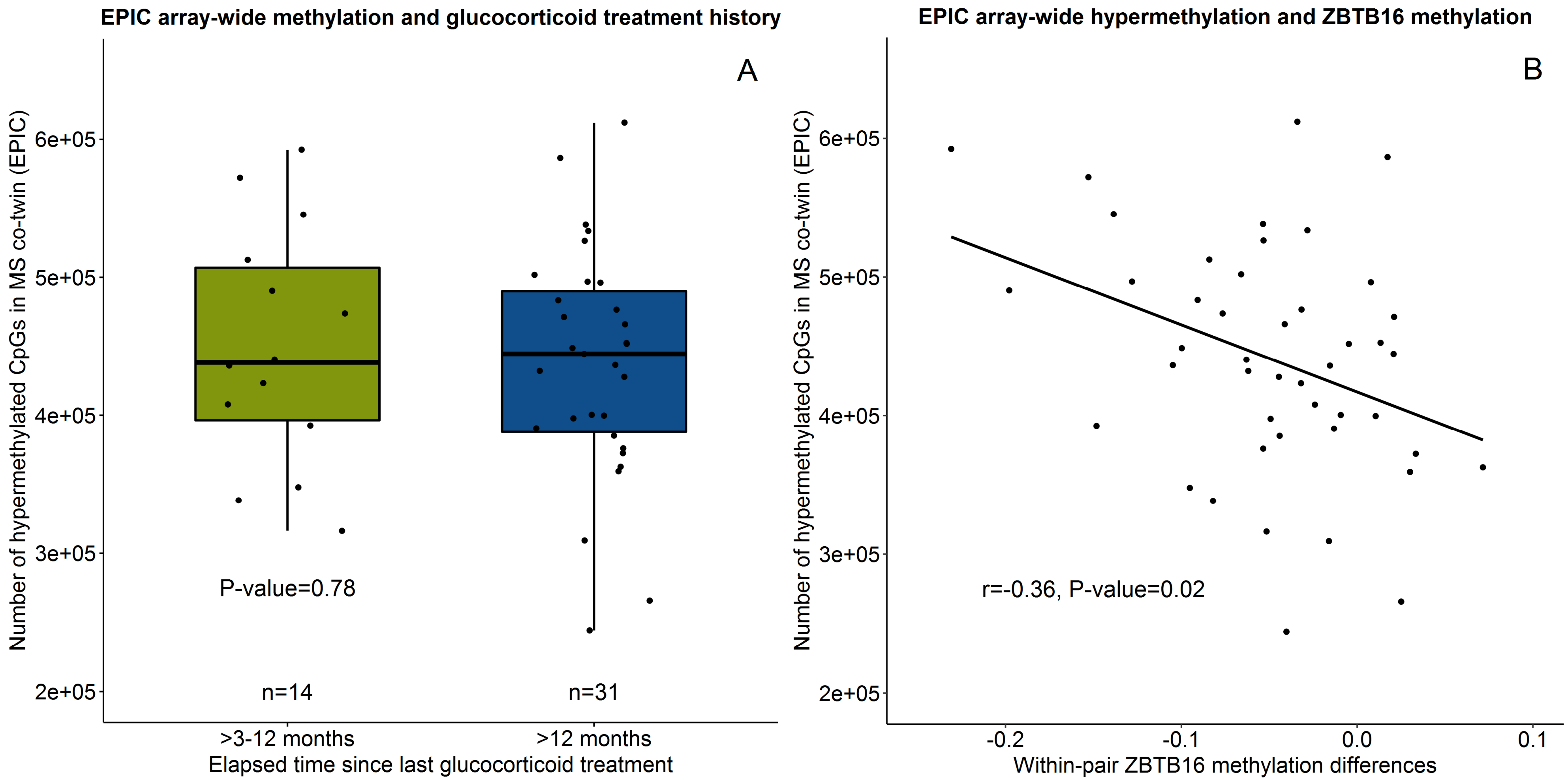

Supplementary Figure 18. EPIC-array wide hypermethylation, glucocorticoid treatment history and *ZBTB16* methylation (n = 45 twin pairs). (A) Number of hypermethylated CpGs in the MS-affected co-twins and elapsed time since last glucocorticoid treatment in the MS-affected MZ co-twin. Data are presented as Tukey boxplots. P-value = non-parametric two-tailed Wilcoxon rank-sum test result. **(B)** Number of hypermethylated CpGs in the MS-affected co-twins plotted against the within-pair methylation differences of the *ZBTB16* DMP (cg25345365, TDBS). r = Pearson's correlation coefficient with P-value, within-pair methylation difference = MS-affected MZ co-twin – clinically non-affected MZ co-twin. Source data are provided as a Source Data file. Boxplots represent the median (central line), the interquartile range or IQR (bottom and top of the box), and 1.5 times the IQR (whiskers).

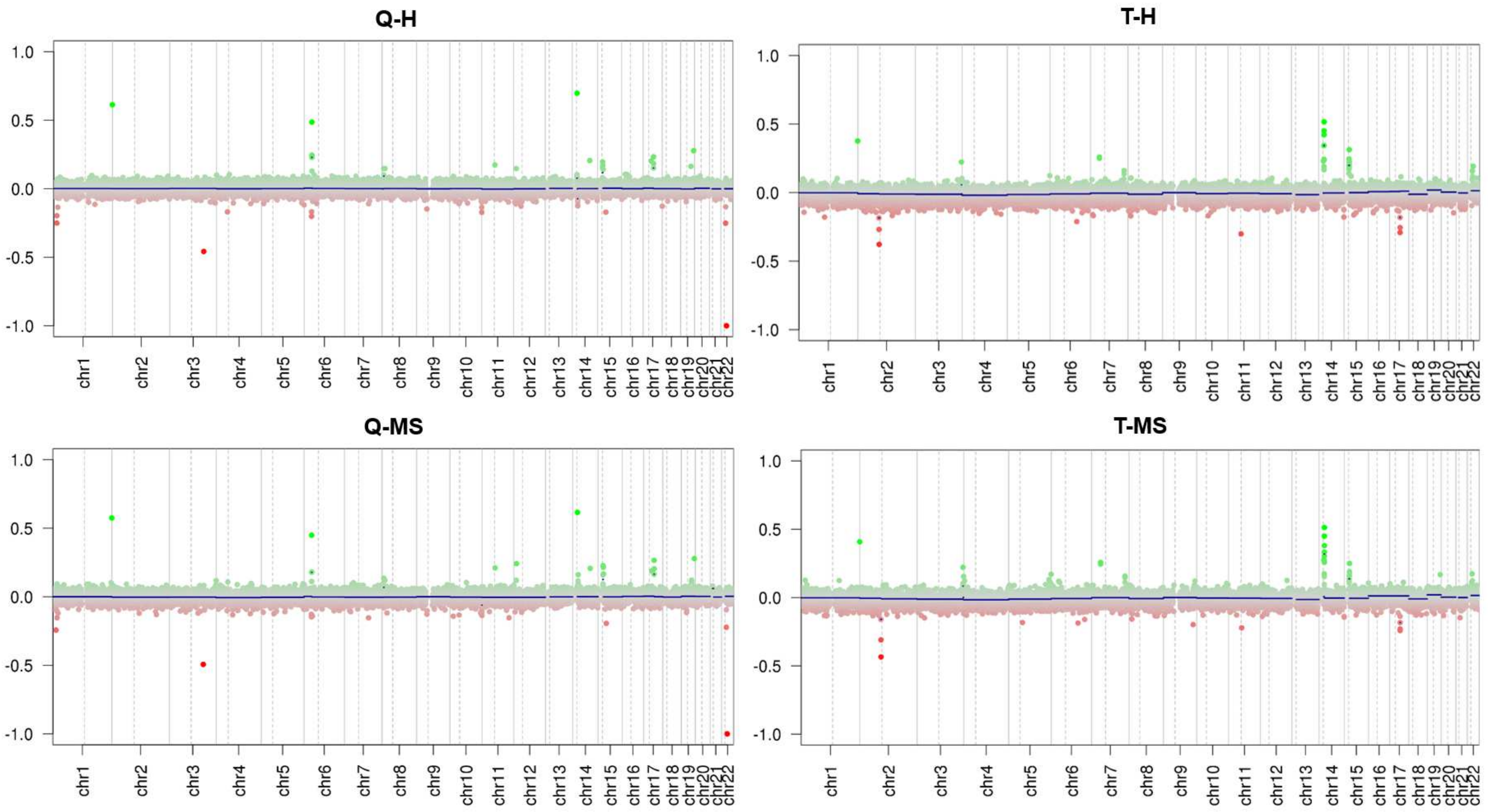

Supplementary Figure 19. An example of copy number plots generated from the Infinium MethylationEPIC BeadChip data of non-affected co-twins (Q-H & T-H) and their MS-affected MZ co-twins (Q-MS & T-MS). Gains are indicated in green and losses in red. MZ co-twins show very similar copy number profiles and no within-pair chromosomal gains and losses (defined as absolute segment mean threshold ≥0.3) were observed. Labels indicate: Pair ID - Disease status (i.e. MS = MS-affected MZ co-twin, H = clinically non-affected MZ co-twin).

```
>chr11:114049864-114050363
GTTGTTTTCCTTTCAGCCAGGATTTGCTCACCTGCTTTCCCTTTTCCCTCTTCCCTGAAT
CTGTGTATCTTCCAGAAACTCTGGTTCTGGATCAGCTTTTTGTTTTGTTGACAAAGGAGA
AAGAGCAAGAGAGAGAGCTAGAGAGAGAGAGAGAGAGAGCCTTCAAGCTCCTCTCTGGGA
GTACACATCTCCTTGAGGGAAAGAACACACAGTGCCGGCCTTTGGAATTGGCAGCCAGTG
TGCTGTTCTCCGTCTGATAAGAGGTACTGTAAATAAAACTGTACACCATGGCCTGTTGTA
AAATGCCCTGCGTCTGTACTCATTGTTCTGACAGCTTATGCTTTTTTTGGGTCTGCTGTT
TTGGTACACTCTGTACTTCCTTATGTAAGCAGGCGTGCAGATCTCATCAGAACATTCAAG
ATGTTTATTTTAAAATCTCAAGGAATTTTGAAAAAAAGGACACACCACTCAACATTAGAT
GCTGGCAAACATTAGGTGTT
```

Supplementary Figure 20. Genomic sequence of the *ZBTB16* DMP (cg25345365) region. The cg25345365 CpG is marked in green and the other CpGs in this region are marked yellow. The consensus GRE downstream half-sites (TGTTCT) are in bold and marked blue. The genomic position of the forward and reverse primers used to generate the amplicon for the TDBS analysis are bold and underlined. Genome coordinates are human genome build GRCh37/hg19.

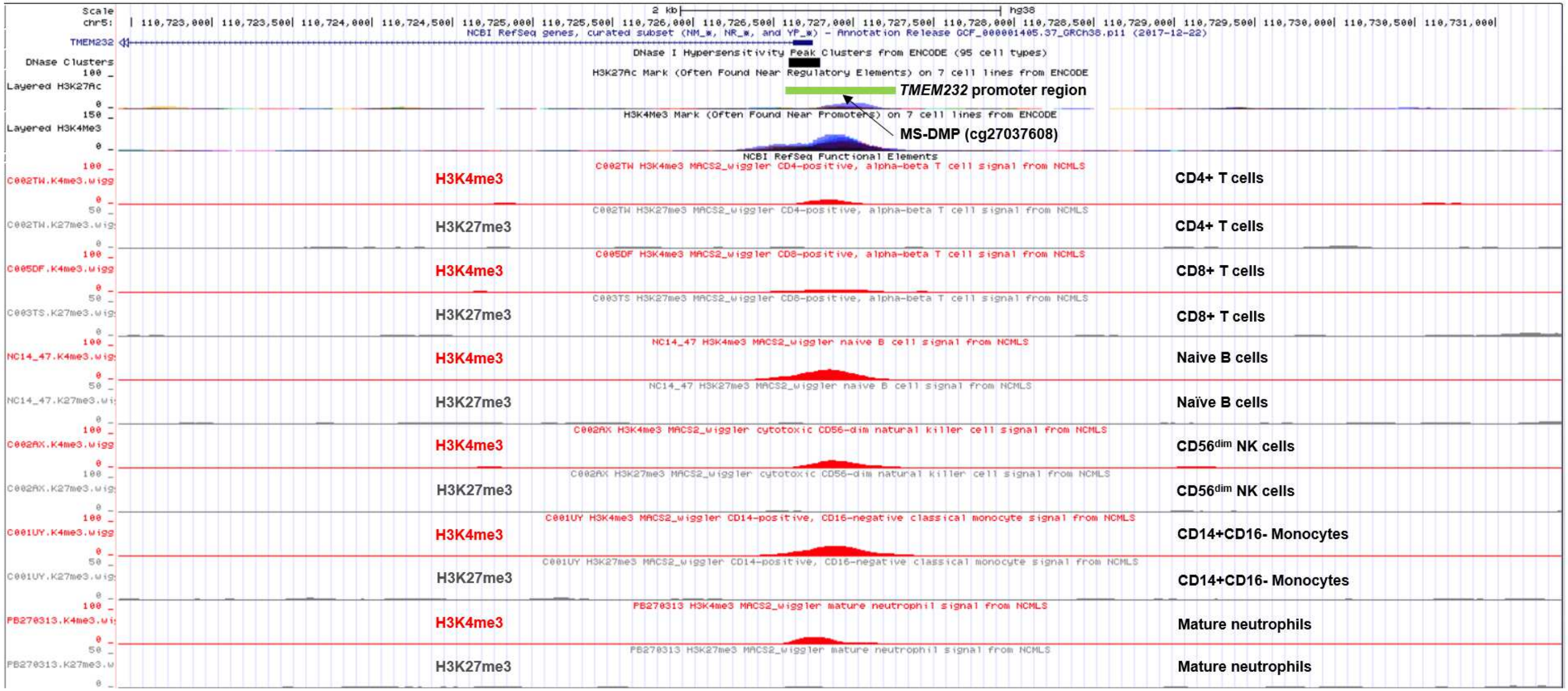

Supplementary Figure 21. Status of the active H3K4me3 and repressive H3K27me3 chromatin marks at the *TMEM232* promoter region in different immune cell types from BLUEPRINT samples.[15] Note that all genome coordinates are based on human genome build GRCh38/hg38. MS-DMP = MS-associated differentially methylated position.

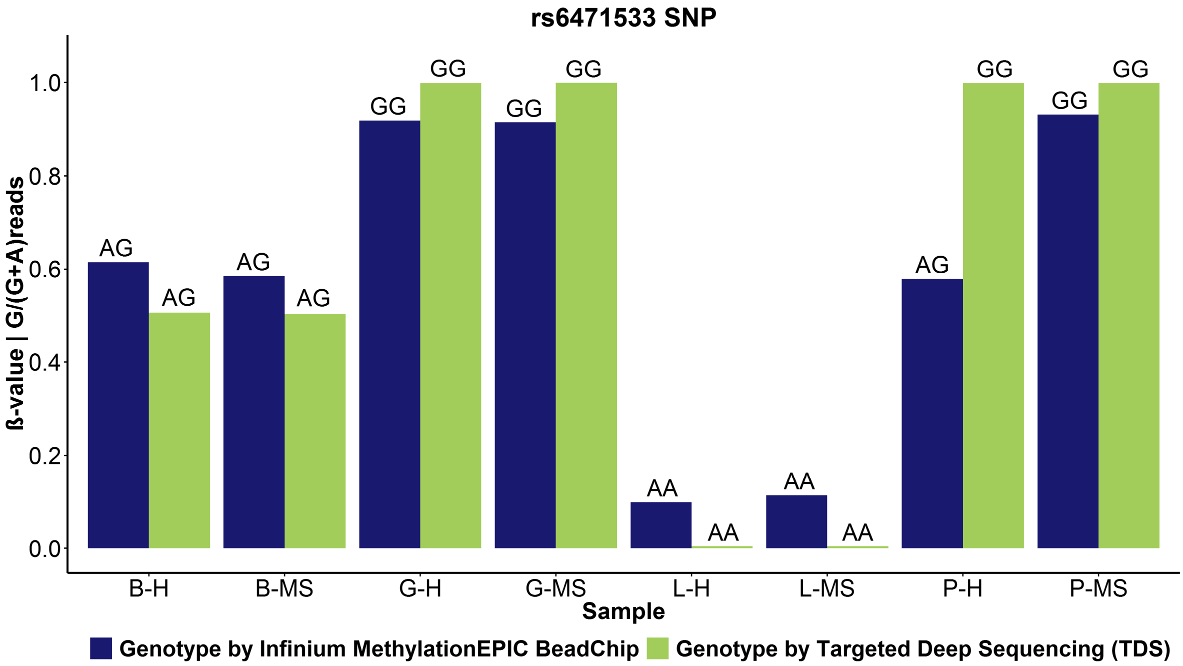

Supplementary Figure 22. Validation of the rs6471533 SNP using targeted deep sequencing (TDS) in pair P, which shows a discordant genotype for the rs6471533 SNP on the Infinium MethylationEPIC BeadChip (n = 4 twin pairs). The rs6471533 SNP was also genotyped using TDS in pair B heterozygous for the rs6471533 SNP, pair G homozygous for the G allele and pair L homozygous for the A allele, according to the Infinium MethylationEPIC BeadChip. Hence, TDS reveals that both co-twins of pair P are homozygous G allele carriers, and the genotypes of the other pairs agrees with the genotype of the Infinium MethylationEPIC BeadChip.

On the y-axis the Infinium MethylationEPIC BeadChip β-values as well as the TDS results are shown, both represented as the fraction of guanines (0=AA and 1=GG). Labels indicate: Pair ID - Disease status (i.e. MS = multiple sclerosis-affected MZ co-twin, H = clinically non-affected MZ co-twin).

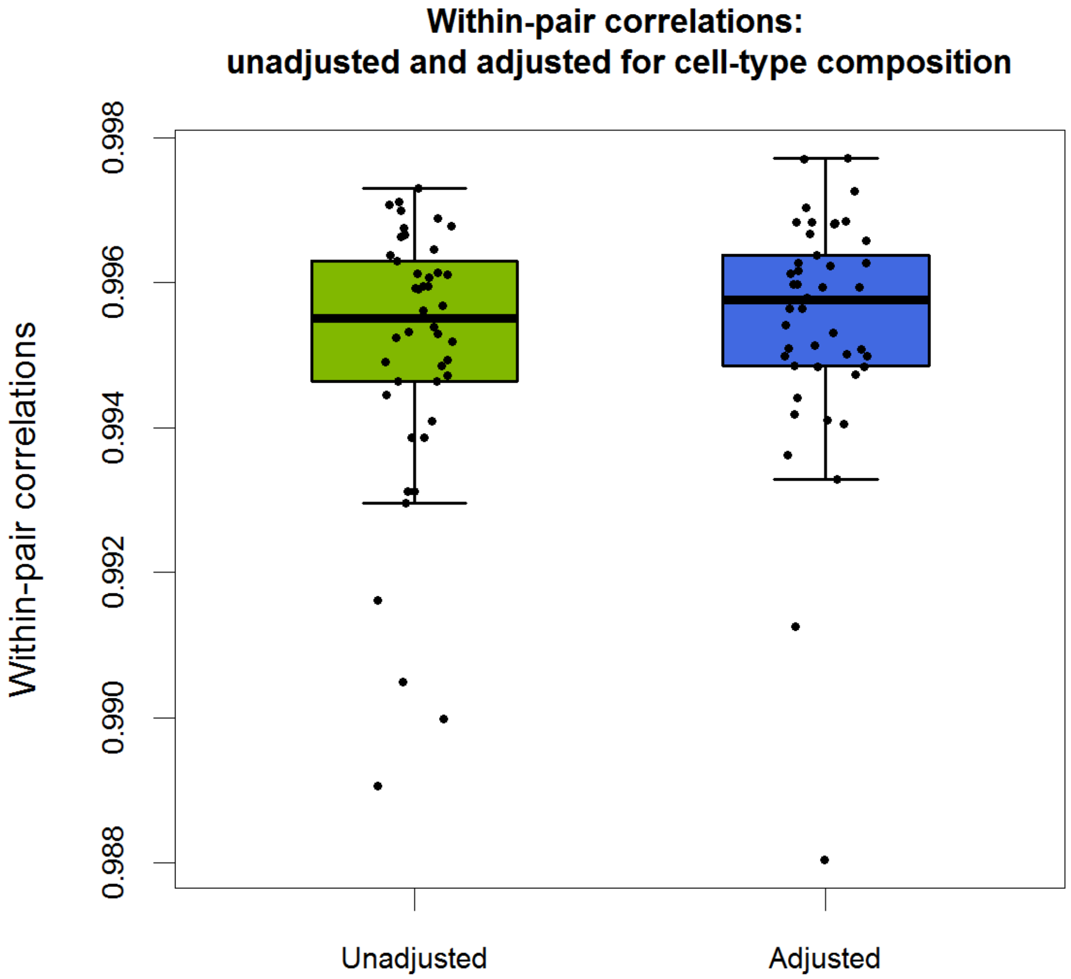

Supplementary Figure 23. Tukey Box-plots of the within-pair Pearson correlation coefficients before and after adjusting the Infinium MethylationEPIC BeadChip data for cell-type composition (n = 45 twin pairs). Boxplots represent the median (central line), the interquartile range or IQR (bottom and top of the box), and 1.5 times the IQR (whiskers).

Supplementary Methods

Bisulfite treatment

From each PBMC sample, 500 ng DNA was treated with bisulfite using the EZ DNA Methylation kit (D5002, Zymo Research), according to the manufacturer's recommendations for the Illumina Infinium assay. The conversion reaction was incubated at 16 cycles of 95°C for 1 min and 50°C for 60 min. For all six MZ twin pairs that were processed in the first batch, the bisulfite controls present on the Illumina Infinium MethylationEPIC BeadChip (EPIC array) showed suboptimal results, i.e., bisulfite conversion controls (I and II) showed moderate intensities at probes which should be at background level. However, the within-pair array-wide Pearson correlation coefficients for these MZ twin pairs were very high and ranged from 0.996 to 0.997, indicating high quality methylation data. We verified the conversion rate of these samples by targeted, deep bisulfite sequencing (TDBS) of a 343-bp region amplified using non-bisulfite-dependent primers (Chr1:202150908-202151251, see **Supplementary Table 12**). TDBS of these samples showed an average conversion rate of 98.6% (SD=0.37%) (minimal coverage >2000 reads), indicating that the EPIC array bisulfite controls are extremely sensitive. Hence, these EPIC array data were used in the downstream analysis. Nevertheless, the bisulfite treatment procedure was adapted by incubating samples in a programmable ThermoQ Metal Bath with a heated lid (Bioer, Hangzhou, China) instead of a Eppendorf Mastercycler (Eppendorf AG, Hamburg, Germany), and TDBS in 16 samples revealed an average conversion rate of 99.7% (SD=0.10%). Accordingly, the other 40 MZ twin pairs were processed using the adapted bisulfite treatment and the EPIC array bisulfite controls showed normal intensities for those samples. Both members of a twin pair were always processed in the same batch.

Estimation of cell-type composition

Cell-type composition of each PBMC sample was estimated with the reference-based method first published by Houseman et al.[6], which employs DNA methylation reference profiles of individual cell types to estimate the cell-type composition of each sample. Several reference-based deconvolution algorithms were compared, including the implementation of the Houseman algorithm in the *minfi* R/Bioconductor package[7], and the standard constrained projection as well as the two non-constrained, reference-based, cell-type deconvolution approaches recently implemented in the EpiDISH R/Bioconductor package[16]. For a subset of samples (n=61) cell-type proportions determined using immunophenotyping were available, which showed the best correlation with the estimates provided by the *minfi* package. Accordingly, the *minfi* estimates were used to adjust the

β-values for cellular composition using linear regression and the residuals were used for downstream analysis. To obtain interpretable, adjusted β-values, the unadjusted mean β-value of each CpG site was added to the residuals. To check the quality of the adjustment, the adjusted β-values were used to recalculate the within-pair correlations. In the final regression model, the proportions of the four major lymphocyte subtypes were included (i.e., CD4+ T, CD8+ T, CD19+ B, and CD56+ NK cells). Myeloid cells (i.e., monocytes and neutrophils) were not included in the model as immunophenotyping data showed that monocyte proportions were not properly estimated and including them resulted in severe adjustment bias in some samples. As a result, **Supplementary Figure 23** shows that the overall within-pair correlations are, as expected, higher after adjusting for cell-type composition.

Cell sorting procedure, WGBS library preparation and sequencing data preprocessing

Whole genome-wide bisulfite sequencing (WGBS) was used to profile CD4+ central and effector memory T cells of four MS-discordant female MZ twin pairs (mean age 43.3 years, discordant for MS >12 years, **Supplementary Table 13**). Of one pair, the MS-affected co-twin had been treated very recently with GCs at the time of blood collection (but never received any immune-modulating therapy), while the MS-affected co-twins of the other three pairs had not received GCs or other immune-modulating therapies within at least 12 months prior to blood collection.

Cryopreserved PBMCs were thawed and gently suspended in 10 ml of pre-cooled FACS buffer (PBS, 2% FCS) and centrifuged at 300 g for 10 min at 4°C. Then, one additional washing step was performed. Cells were stained with the following monoclonal antibodies: CD3-AF700 (OKT3, eBioscience, Frankfurt, Germany); CD4-Pacific-Blue (S3.5, Molecular Probes, Invitrogen, Karlsruhe, Germany); CD8-PerCP (SK1, BioLegend, Fell, Germany); CD45RO-FITC (UCHL1, eBioscience); and CCR7-APC (3D12, eBioscience) on ice for 30 minutes. Cells were then sorted using a FACSAria Fusion flow cytometer (BD Biosciences, Heidelberg, Germany) to selectively collect antigen-experienced CD4+ T cells by excluding dead cells, naive CD45RA+CCR7+ T cells, and CD8+ T cells.

WGBS libraries were prepared using a tagmentation-based protocol similar to that described by Weichenhan et al.[17]. Briefly, fresh frozen primary CD4 cell pellets (each 20,000-200,000 cells) were thawed in 50-100 µl of 1.1x TD buffer (Illumina) supplemented with 6 µl Protease (1 mg/ml; Qiagen) and incubated in a thermomixer at 55°C for 3 h followed by 20 min at 75°C. DNA was quantified using the Qubit HS-DNA kit (Thermo Fisher Scientific, Waltham, USA). From each sample the volume corresponding to 50 ng DNA was transferred in a new 1.5-ml tube and 1x TD buffer was added to a total volume of 47.5 µl. Then, the DNA was tagged with 2.5 µl of Tn5 from

the Nextera library preparation kit (Illumina) by incubation for 5 min at 55°C. After purification with the MinElute kit (Qiagen) and final elution with 10 µl EB buffer, gaps were repaired by adding 2 µl of 10x CutSmart buffer (NEB, Ipswich, USA), 3 µl of dNTPs (2.5 mM each), 5 U Klenow exo- (NEB) and incubating for 1 h at 30°C. Bisulfite conversion was performed with the EZ Methylation Gold Kit (Zymo Research) with a final 10 µl elution volume. Indexing library enrichment PCR was performed in 40 µl reactions with 1x HotStartBuffer (Qiagen), 0.25 mM of each dNTP, 0.3 µl ssDNA Binding Protein (Affymetrix, Santa Clara, USA), 100 nM of each primer (reverse primer contains sample-specific DNA barcode), 4 U HotStartTaq DNA polymerase (Qiagen), and 10 µl bisulfite-converted DNA. DNA was denatured at 95°C for 15 min, followed by 12 cycles of 30 sec at 95°C, 2 min at 53°C, and 1 min at 72°C, and a final extension step of 7 min at 72°C. Reactions were purified using 0.8x volume AMPure XP Beads (Beckman Coulter, Brea, USA) and eluted in 10 µl Elution Buffer (Qiagen). Library fragment distributions were checked on the Agilent Bioanalyzer (Agilent, Santa Clara, USA).

The WGBS libraries were sequenced in a 100-bp paired-end HiSeq2500 run (Illumina) using custom sequencing primers. After adapter trimming using Trimmomatic v0.36[18], the read pairs were aligned to the human reference genome (GRCh37) using *bwa-meth* v0.2.0[19], which is a wrapper of the BWA-MEM1 alignment algorithm suited for bisulfite sequencing data. PCR duplicates were removed using the MarkDuplicates tool of the Picard suite v2.5.0-1 (http://broadinstitute.github.io/picard). Methylation levels of the CpG cytosines were determined using MethylDackel v0.2.1 (https://github.com/dpryan79/MethylDackel.git). Of both read mates, 10 base pairs were disregarded from both read ends to eliminate the gap repair bias and methylation bias artifacts. The obtained BED files were loaded in the RnBeads package, which aggregated for each CpG the methylation information of both strands. The coverage statistics of the samples are summarized in **Supplementary Table 13**.

Supplementary References

1. Kular, L. *et al.* DNA methylation as a mediator of HLA-DRB1*15:01 and a protective variant in multiple sclerosis. *Nat Commun* **9**, 2397 (2018).
2. Beecham, A.H. *et al.* Analysis of immune-related loci identifies 48 new susceptibility variants for multiple sclerosis. *Nat Genet* **45**, 1353-60 (2013).
3. Sawcer, S., Franklin, R.J. & Ban, M. Multiple sclerosis genetics. *Lancet Neurol* **13**, 700-9 (2014).
4. Yu, G., Wang, L.G. & He, Q.Y. ChIPseeker: an R/Bioconductor package for ChIP peak annotation, comparison and visualization. *Bioinformatics* **31**, 2382-3 (2015).
5. Teschendorff, A.E. *et al.* DNA methylation outliers in normal breast tissue identify field defects that are enriched in cancer. *Nat Commun* **7**, 10478 (2016).
6. Houseman, E.A. *et al.* DNA methylation arrays as surrogate measures of cell mixture distribution. *BMC Bioinformatics* **13**, 86 (2012).
7. Aryee, M.J. *et al.* Minfi: a flexible and comprehensive Bioconductor package for the analysis of Infinium DNA methylation microarrays. *Bioinformatics* **30**, 1363-9 (2014).
8. Wu, H. *et al.* Detection of differentially methylated regions from whole-genome bisulfite sequencing data without replicates. *Nucleic Acids Res* **43**, e141 (2015).
9. Price, A.L., Eskin, E. & Pevzner, P.A. Whole-genome analysis of Alu repeat elements reveals complex evolutionary history. *Genome Res* **14**, 2245-52 (2004).
10. Aranyi, T., Varadi, A., Simon, I. & Tusnady, G.E. The BiSearch web server. *BMC Bioinformatics* **7**, 431 (2006).
11. Liguori, M. *et al.* Age at onset in multiple sclerosis. *Neurol Sci* **21**, S825-9 (2000).
12. O'Connor, P. & Canadian Multiple Sclerosis Working, G. Key issues in the diagnosis and treatment of multiple sclerosis. An overview. *Neurology* **59**, S1-33 (2002).
13. Hacisuleyman, E., Shukla, C.J., Weiner, C.L. & Rinn, J.L. Function and evolution of local repeats in the Firre locus. *Nat Commun* **7**, 11021 (2016).
14. Izuogu, O.G. *et al.* Analysis of human ES cell differentiation establishes that the dominant isoforms of the lncRNAs RMST and FIRRE are circular. *BMC Genomics* **19**, 276 (2018).
15. Stunnenberg, H.G., International Human Epigenome, C. & Hirst, M. The International Human Epigenome Consortium: A Blueprint for Scientific Collaboration and Discovery. *Cell* **167**, 1145-1149 (2016).
16. Teschendorff, A.E., Breeze, C.E., Zheng, S.C. & Beck, S. A comparison of reference-based algorithms for correcting cell-type heterogeneity in Epigenome-Wide Association Studies. *BMC Bioinformatics* **18**, 105 (2017).
17. Weichenhan, D. *et al.* Tagmentation-Based Library Preparation for Low DNA Input Whole Genome Bisulfite Sequencing. *Methods Mol Biol* **1708**, 105-122 (2018).
18. Bolger, A.M., Lohse, M. & Usadel, B. Trimmomatic: a flexible trimmer for Illumina sequence data. *Bioinformatics* **30**, 2114-20 (2014).
19. Pedersen, B.S., Eyring, K., De, S., Yang, I.V., Schwartz, D.A. Fast and accurate alignment of long bisulfite-seq reads. *arXiv:1401.1129 [qbio.GN]*.
